# Water, sanitation, and hygiene in schistosomiasis control: A scoping review of evidence gaps across transmission pathways

**DOI:** 10.1371/journal.pgph.0006916

**Published:** 2026-07-30

**Authors:** Laura Braun, Yael Velleman, Anne Straily, W. Evan Secor

**Affiliations:** 1 Environmental Health Group, London School of Hygiene and Tropical Medicine, London, United Kingdom; 2 Policy and Innovation Team, Unlimit Health, London, United Kingdom; 3 Division of Parasitic Diseases and Malaria, US Centers for Disease Control and Prevention, Atlanta, Georgia, United States of America; 4 Division of Parasitic Diseases and Malaria, US Centers for Disease Control and Prevention, Atlanta, Georgia, United States of America; Babcock University, NIGERIA

## Abstract

Schistosomiasis is a water-based disease transmitted through contact with freshwater contaminated by cercariae from infected snails. Transmission is shaped by access to water, sanitation, and hygiene (WASH) services, human behaviour, and environmental factors. While several systematic reviews have examined WASH-schistosomiasis links, a synthesis of emerging evidence and identification of knowledge gaps across the transmission pathways is needed. This scoping review aimed to map WASH-related risk factors linked to schistosomiasis transmission, to assess alignment with a schistosomiasis-specific conceptual framework, and to characterise methodological features of the evidence base, including diagnostics and WASH measurement. We searched five databases, supplemented by a call for papers, yielding 2915 de-duplicated records; 181 were included (160 observational, 21 interventional studies). We developed a conceptual framework to map WASH-related transmission pathways and identify evidence gaps. Our analysis reveals a growing body of research on WASH access and practices, yet key gaps persist. Observational studies mostly examined domestic, recreational, and occupational water contact, with limited attention to transmission pathways such as agricultural use of faecal waste; most relied on simple access measures, rarely assessing WASH quality, functionality, or sustained use, limiting the ability to translate findings into effective service delivery strategies. Among interventional studies, 43% focussed on water access, 24% implemented integrated WASH and health education, and 19% sanitation, hygiene, and education. Over 90% reported reduced infection, but few assessed sustained uptake or environmental impacts. Limitations include lack of species-specific reporting, inconsistent WASH definitions, and insensitive diagnostics. Future studies would benefit from adopting standardised WASH definitions and transmission-specific frameworks, report outcomes by species and infection intensity, and design interventions with explicit theories of change addressing transmission pathways. Intervention studies should assess fidelity, sustained uptake, and community-level coverage alongside infection outcomes, with follow-up to capture reinfection dynamics, strengthening evidence for integrating WASH with preventive chemotherapy in elimination programmes.

## Introduction

Schistosomiasis, also known as Bilharzia or Snail Fever, is a parasitic disease caused by *Schistosoma* spp. Transmission occurs when eggs from infected persons enter freshwater sources through urine (for *Schistosoma haematobium*) or faeces (for *S. mansoni* and all other species). If suitable freshwater snail species are present, the parasites develop inside them before being released into the water as larvae (cercariae), which can penetrate human skin during water contact. Once inside the body, the parasites mature and produce eggs, some of which are excreted, continuing the cycle. Schistosomiasis can lead to diarrhoea, haematuria, anaemia, developmental delays, enlargement of liver and spleen, and genital schistosomiasis, often contributing to significant morbidity in endemic regions [1,2].

Schistosomiasis transmission pathways are shaped by the availability, accessibility, affordability, acceptability, and quality of water supply, sanitation and hygiene (WASH) services. Limited access to safe water and inadequate sanitation facilitates the spread of infection, while behaviours such as bathing, swimming, and washing clothes in contaminated water increase exposure risk. Due to its dependence on freshwater bodies and specific snail hosts, schistosomiasis is highly focal in nature.

Previous systematic reviews have examined the relationship between WASH and schistosomiasis. Grimes et al. (2014) found that access to safe water and adequate sanitation were associated with lower odds of both *S. mansoni* and *S. haematobium* infections [3]. Similarly, Freeman et al. (2018) identified an association between sanitation and lower odds of both *S. mansoni* and *S. haematobium* infections, though this held only when comparing any sanitation with no sanitation, rather than improved versus unimproved sanitation [4]. Water treatment processes have been shown to effectively remove or inactivate schistosome cercariae, yet variations in initial water quality and measurement approaches limit study comparability [5].

Beyond sanitation and water treatment, human-water contact behaviours are a critical factor in schistosomiasis transmission. A meta-analysis by Reitzug et al. found that individuals in communities with ≥10% prevalence who engaged in water contact had a threefold higher infection risk compared to those who did not [6]. However, no associations were observed between the duration or frequency of exposure and infection risk.

### The focus on elimination and background to review

In 2024, schistosomiasis was endemic in 79 countries, with preventive chemotherapy (PC) required in 50 countries [7]. PC with praziquantel, through the World Health Organization (WHO)-managed drug donation programme, has been the primary control strategy for the past two decades. The WHO neglected tropical diseases (NTD) 2021–2030 road map goal is to eliminate schistosomiasis as a public health problem [8], yet over 250 million people, primarily school-aged children, still require preventive chemotherapy [7].

While most control efforts focus on reducing prevalence to below the 10% threshold to reduce the need for mass treatment [9], some countries such as China [10] or St Lucia [11,12] are pursuing transmission elimination through integrated healthcare and environmental interventions. The process for verifying interruption of transmission is under development.

Significant barriers to elimination persist. PC alone is insufficient to break transmission, requiring complementary strategies [8]. Snail control can reduce intermediate host populations but can pose environmental risks, and snails can rapidly recolonise or repopulate through self-fertilisation [13,14]. Similarly, asexual reproduction of schistosomes within snails results in amplification of infectious parasites in water bodies. The percentage of infected snails is often disproportionately small compared to the prevalence of human infection.

Despite evidence from systematic reviews indicating that improved WASH access is associated with reduced schistosomiasis prevalence, simply providing access to water and sanitation infrastructure does not necessarily translate into reduced infection risk, largely due to the persistent interaction between at-risk populations and contaminated surface water. Furthermore, achieving sustained behaviour change to reduce exposure is challenging [15], given the focal nature of schistosomiasis transmission, the long latency between infection and the development of severe morbidity, and the persistence of misconceptions shaped by cultural beliefs and practices.

Efforts to achieve the targets set out in the 2021–2030 WHO NTD road map [8] have renewed attention to the role of WASH in interrupting transmission of schistosomiasis. In 2021, the WHO Department for the Control of Neglected Tropical Diseases convened the Technical Advisory Group (TAG) on Soil-Transmitted Helminthiasis and Schistosomiasis Control and Elimination to provide strategic and technical guidance to countries pursuing elimination goals. A dedicated WASH sub-group was formed to strengthen the evidence base linking WASH interventions to transmission dynamics and to inform policy development and research prioritisation.

The WASH sub-group agreed that the biological and epidemiological connections between WASH conditions and schistosomiasis transmission are well recognised. Rather than re-evaluating the magnitude of associations between WASH indicators and infection outcomes, a scoping review approach [16] was decided to be most appropriate to systematically map how WASH-related exposures have been conceptualised and measured in relation to schistosomiasis transmission pathways, with a focus on alignment between exposure definitions and hypothesised mechanisms of effect.

Although WASH is often discussed jointly for schistosomiasis and STH, important differences in transmission biology warrant separate analyses. A companion scoping review on WASH and STH has been published. We developed a schistosomiasis-specific conceptual framework that captures sanitation services and related behaviours, pathways of faecal contamination of freshwater, the role of water services, and distinct drivers of water contact.

By mapping the breadth and structure of available evidence, this review seeks to clarify where empirical support is strong, where measurement approaches lack conceptual coherence, and where critical gaps remain along the transmission pathway. These insights are intended to guide future research design and strengthen the integration of WASH within schistosomiasis control and elimination strategies.

## Methods

**Fig 1:**
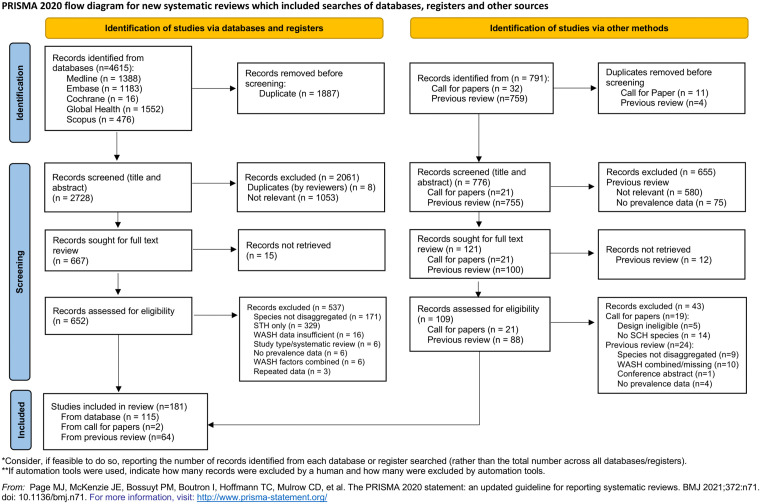
Preferred Reporting Items for Systematic Reviews and Meta- Analyses (PRISMA) diagram demonstrating the records identified, included, and excluded, with reasons for exclusion. The flow diagram structure is from the PRISMA guideline [18].

The protocol for this scoping review was registered on the Open Science Framework registries website on 4 October 2022 [17], and was conducted in accordance with the PRISMA guidelines. The PRISMA diagram is shown in Fig 1, and a PRISMA checklist is included in the supplementary information (S1 File).

### Search strategy

**Fig 2:**
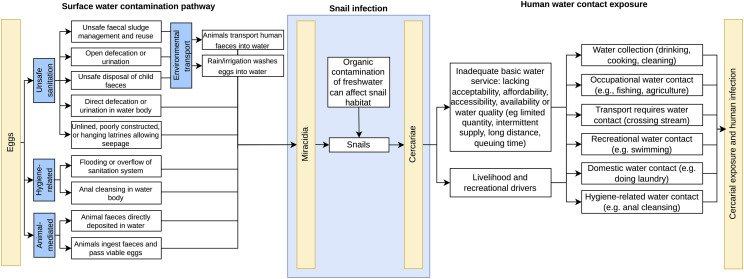
WASH-specific conceptual framework for schistosomiasis transmission.

The search strategy includes database searches, a call for papers, and results from a previous review [3]. We searched five databases (Medline, Embase, Cochrane, Global Health, and Scopus) using the search terms in the supplementary information (S1 Table). The search strategy was informed by a conceptual framework (Fig 2) setting out all likely transmission pathways related to WASH practices and infrastructure. Searches were conducted in English in September 2022 and updated in May 2025. Additionally, a call for papers was shared in March 2022 through various networks and channels, including the NTD NGO network membership list, the Sustainable Sanitation Alliance (SuSanA) Forum, the WHO PHE newsletter and through social media. The reference lists of included studies and identified relevant systematic reviews were hand-searched for additional relevant studies.

The literature search focused on studies published on or after 1 January 2014 to capture studies published since the last comprehensive systematic review and meta-analysis by Grimes et al [3]. All included studies, as well as studies excluded by the authors for not providing sufficient data for the meta-analysis despite being on schistosomiasis and WASH, were screened based on the inclusion criteria of this review.

### Screening and data extraction

Search results were de-duplicated in the reference management system Endnote [19] and uploaded to Rayyan, a systematic literature review application [20]. Titles and abstracts were double screened, split equally among four reviewers (YV, ES, AS, LB). Disagreements were resolved through discussion among reviewers. Full texts of articles meeting the inclusion criteria were retrieved and double screened by two reviewers, with the list of articles and decisions noted in Microsoft Excel. A pre-specified data extraction sheet was used to extract data from included studies (including those from Grimes et al [3]), listing study metadata (author, year, title, country, study design, diagnostic method, setting (urban/rural), number, age and details of participants, study aims) and data items (variables) of interest. Given that schistosomiasis and STH are often studied simultaneously, the search, abstract review and full text review were conducted jointly for schistosomiasis and STH. The two diseases were then separated at the data extraction phase.

### Inclusion criteria

Studies with an abstract in English, French and Spanish published in peer-reviewed journals were considered. Both observational and intervention studies were included if they quantified WASH-related exposure and schistosomiasis infection (prevalence or intensity of infection). There were no limits to diagnostic methods used. Studies that reported genus-level as opposed to species-level infection, as well as studies combining infection measures for schistosomiasis with STH infections (e.g., prevalence of any intestinal helminth), were excluded. Studies linking WASH to parts of the lifecycle besides human infection (e.g., snail infection, presence of cercariae in water) were also excluded.

## Results

The review identified a total of 4615 records via the database search, 32 via the call for papers, and 759 from the review by Grimes et al [3] (Fig 1). Following de-duplication, 2728 titles and abstracts identified via databases were screened, and 667 by full text. Most studies were excluded as they only reported STH infections, did not disaggregate results from STH infections or by *Schistosoma* species, or combined multiple WASH variables (e.g., “access to safe water and sanitation”). A total of 115 studies were included in this review from the database search. Of the 32 studies identified through the call for papers, 2 are included, with most being excluded as they focused on STH infections. Of the 759 identified from the previous review, 64 studies are included. Overall, 181 studies are included in this review - 115 from the literature search, 2 from the call for papers and 64 from the previous review. Of these, 21 studies are intervention trials and 160 are observational studies.

### Observational studies

Among observational studies (160/181), 75% (n = 120/160) were conducted in Africa, 16% (n = 25) in South America, and 9% (n = 15) in Asia (S2 Table and S3 Table). Ethiopia (n = 29/160) was the country where most studies were conducted, followed by Nigeria (27/160) and Brazil (25/160). In Asia, most studies were conducted in Yemen (n = 6). 65% of observational studies (105/160) were conducted in rural settings, 12% in peri-urban or urban settings (16/160) and 15% in both rural and urban (24/160), with 8% not indicating setting type (13/160). On average, studies included 1604 participants, and in total, 258,225 participants. Half of the studies included only young and school-aged children (81/160) (<19 years old), with most focussing on the 6–15-year age range. A total of 46% of studies focussed on adults and children (74/160), and 3% (n = 5) included only adults. More than half of observational studies reported *S. mansoni* infections (88/160), followed by *S. haematobium* (56/160), with 5% of studies reporting both *S. mansoni* and *S. haematobium* (8/160). Only seven studies on *S. japonicum* and two studies on *S. mekongi* were included. Supplementary Information (S2 Table and S3 Table) report the extracted data and citation for each observational study. Most observational studies evaluated both intensity of infection and prevalence, but only 12% (19/160) analysed the relationship between intensity (i.e., egg output) and WASH-related factors.

**Fig 3:**
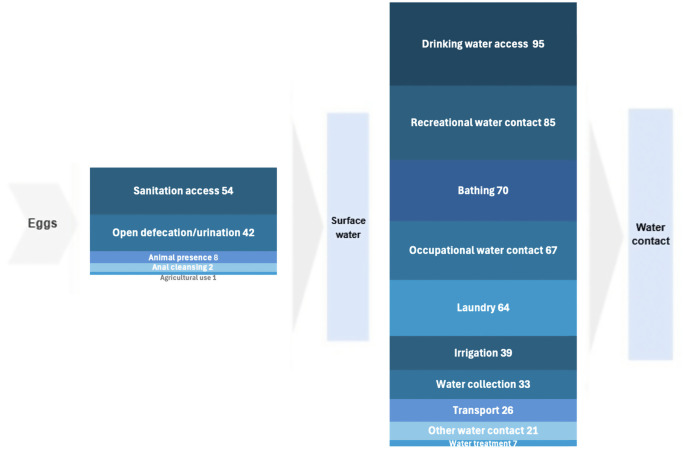
Schistosomiasis transmission pathways by study frequency. Diagram showing the number of studies reporting schistosomiasis transmission routes from parasite egg contamination sources (left) through surface water (centre) to human water contact exposures (right). Box heights are proportional to the frequency of study reporting for each pathway. “Other water contact” includes unspecified reason for surface water contact.

**Fig 4:**
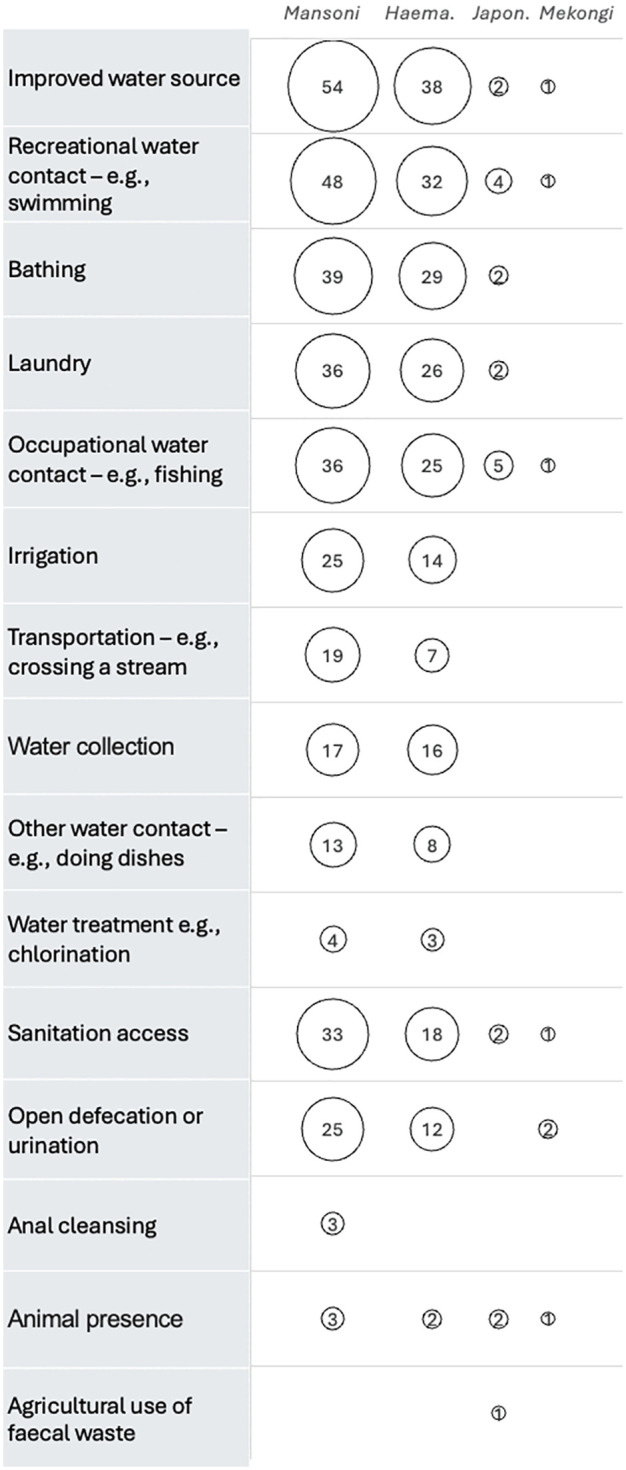
Evidence gap map presenting the number of studies reporting on WASH-related risk factors, irrespective of whether an association was found. Circle numbers and size correspond to the number of studies.

Faecal contamination and sanitation pathways were examined across included studies, with sanitation access (n = 54) and open defecation or urination (n = 42) being the most frequently analysed risk factors (Fig 3). Overall, the association of access to sanitation facilities and schistosomiasis infection was assessed in 30–35% of studies for all species except *S. mekongi* (Fig 4). Additional sanitation-related variables included animal presence, anal cleansing and use of faecal waste in agriculture, which all remained rarely studied across all species. Definitions of sanitation facilities varied greatly across studies (e.g., “latrine,” “toilet,” “latrine with slab connected to septic tank”), and most studies only reported on household-level access rather than sanitation facilities in schools or workplaces where people may spend significant time.

Water contact exposure routes were extensively documented across studies (Fig 3). Drinking water access was the most frequently analysed pathway (n = 95), with 50% of *S. haematobium* and *S. mansoni* studies analysing this risk factor (Fig 4), reflecting the water-based nature of schistosomiasis transmission. However, water sources were defined with varying specificity (e.g., “safe water source” vs. “household water”), and comparisons typically contrasted household- or community-level improved sources (e.g., borehole, piped water) with surface water (e.g., rivers, lakes), making it difficult to determine schistosomiasis risk. Additionally, most studies only assessed primary or drinking water sources, omitting potential contact with alternative unsafe water sources. Recreational water contact (n = 85), bathing (n = 70), occupational water contact (e.g., fishing, rice farming, irrigation, animal/tool washing (n = 67)), and laundry activities (n = 64) were also frequently studied (Fig 3). Transportation by crossing streams or entering canoes and boats was included in 26 studies focussed on either *S. mansoni* or *S. haematobium*. Water collection (n = 33), unspecified water contact activities (n = 21), and water treatment (n = 7) were less frequently examined. S. japonicum studies primarily reported on domestic and occupational water activities, while the two included S. mekongi studies analysed occupational and recreational water contact. Overall, water sources and contact activities were often defined with limited specificity, making it difficult to assess schistosomiasis risk consistently across studies.

### Interventional studies

In total, 21 interventional studies were included in this review (Table 1), conducted between 1961–2017. Four studies were implemented in China, two each in Kenya, South Africa, Tanzania, Sudan and Brazil, and one each in St Lucia, Lao, Côte d’Ivoire, Ghana, Nigeria, Ethiopia and Zimbabwe. All but one study in China [21] were conducted in rural settings. All studies published after 1976 provided treatment with praziquantel as part of the intervention. Interventions focussed on improving safe access to water made of 43% of studies (9/21) and included water supply for domestic and recreational activities. Integrated interventions including WASH and health education were implemented in 5 studies (24%). Four studies, all from China, implemented One Health measures alongside integrated WASH and health education interventions, aiming to interrupt disease transmission by mechanising agriculture and restricting and treating livestock to prevent environmental contamination, engineering safe water and sanitation infrastructure, and providing health education. Three studies focussed on sanitation, hygiene and education. Interventions were implemented across domestic, school and occupational settings, e.g., agricultural fields. Study duration ranged between 9 months and 12 years, with more than 90% of studies (19/21) reporting a reduction in prevalence or intensity of infection following the intervention, compared to baseline or a control.

**Table 1:** Description of included intervention studies. All studies published after 1976 included chemotherapy with praziquantel.

	Author	Study period	Country	Setting	Age of participants (years)	Number of participants (Int/Cont)	Description of intervention	Intervention duration	Species	Reduction in prevalence or re-infection
Water-focussed interventions	Pitchford 1970 [22]		South Africa	Rural	5–9	277	Paddling pool, laundry station, fencing water bodies, cover canals	5 years	*S. mansoni & S. haematobium*	✓
	Jordan et al, 1975 [23]	1968 - 1972	St Lucia	Rural	0–14	658/815	Household piped water supply, health education	2 years	*S. mansoni*	✓
	Tameim et al, 1985 [24]	1980 - 1983	Sudan	Rural	SAC	7,910	Community-level safe water supply (slow sand filter, pump, storage tank), boreholes, snail control	3 years	*S. mansoni & S. haematobium*	✓
	el Kholy et al, 1989 [25]	1984 - 1987	Kenya	Rural	SAC	5,896	Well construction	3 years	*S. haematobium*	╳
	Coura-Filho et al, 1996 [26]	1980 - 1992	Brazil	Both	All	998	Water main installed serving 90% of the district	12 years	*S. mansoni*	✓
	Kosinski et al, 2012 [27]	2008 - 2010	Ghana	Rural	8–22	247	Water recreation areas: concrete pool supplied by a borehole well and gravity-driven rainwater collection system	2 years	*S. haematobium*	✓
	Lee et al, 2015 [28]	2009 - 2011	Sudan	Rural	1st, 3rd and 5th graders	10,000 samples among 78,615 students	Provision of water treatment plant (providing ~ 20L per person per day), health education	9 months	*S. mansoni & S. haematobium*	✓
	Mogeni et al, 2020 [29]	2007 - 2008	South Africa	Rural	9 to 14+	333	Piped water	7 years	*S. haematobium*	✓
	Ekanem et al, 2017 [30]	2009 - 2017	Nigeria	Rural	5–14	Before/after 443/380	Potable water at a fee of 1–3 cents/20L from borehole	8 years	*S. haematobium*	✓
Comprehensive WASH and health education	Barbosa et al, 1971 [31]	1961 - 1968	Brazil	Rural	0–14	1,433	Household latrines, community laundry station, drinking water taps, showers, dug wells and hand pumps, community health education. Limited medical treatment with antimony injections.	7 years	*S. mansoni*	✓
	Chandiwana et al, 1988 [32]	1986 - 1987	Zimbabwe	Rural	All	578	Community-level boreholes, latrines by fields, canals lined with concrete, health education	2 years	*S. mansoni & S. haematobium*	✓
	Poggensee et al, 2005 [33]	1996 - 2002	Tanzania	Rural	6–18	1,058	School drinking water, school toilets, household pit latrines, health education	6 years	*S. haematobium*	✓
	Freeman et al. 2013 [34]	2007 - 2009	Kenya	Rural	7–13	470/445	Hygiene promotion, water treatment technology, sanitation infrastructure, handwashing and drinking water storage containers, 1-year supply of POU water treatment products. Teachers & parent trained in hygiene, water storage, sanitation maintenance, building school latrines	10 months	*S. mansoni*	╳
	Mengistu et al 2024 [35]	2018 - 2025	Ethiopia	Rural	All	Total 6661.Intervention arms: 584/1636/ 2203Control arm: 2238	Construction of deep and shallow water wells and public taps. Mobilisation of people to build and use latrines, community led total sanitation. Other intervention arms implemented the One WASH National Programme.	3 years (results up to phase 2)	*S. mansoni & S. haematobium*	✓
WASH, health education, One Health	Wang et al, 2009 [36]	2002 - 2007	China	Rural	5–65	375	Removing cattle from snail-infested grasslands, providing farmers with mechanised farm equipment, public and household wells, building latrines with septic tank and biogas pools, providing boats with faecal-matter containers, implementing intensive health-education program	5 years	*S. japonicum*	✓
	Sun et al, 2011 [10]	2003 - 2008	China	Rural	6–60	305,719	Replace cattle with machines. Domestic animals raised in pens to reduce grassland contamination. Household tap water, faecal- matter containers for mobile boat fishermen, construction of public latrines with septic tanks, molluscicide, health education. PZQ treatment of livestock	3 years	*S. japonicum*	✓
	Ai, 2013 [21]	2004 - 2012	China	Urban	All	128,374	Household latrines improved, biogas pools were built, river and riverbanks were protected, cattle were eliminated, pond were dug, ditches were hardened, safe drinking water projects were constructed. PZQ treatment of animals.	8 years	*S. japonicum*	✓
	Liu et al, 2017 [37]	2005 - 2014	China	Rural	all ages	7,268,138 humans and 840,845 bovines	Replace bovines with machines, access to clean water, construction of public toilets and household latrines, snail control, health education, PZQ treatment of bovines	10 years	*S. japonicum*	✓
Sanitation&hygiene	Hurlimann et al, 2018 [38]	2011 - 2012	Cote d’Ivoire	Rural	all ages	425/385	Community-led-total sanitation, building latrines, health education	1 year	*S. mansoni & S. haematobium*	✓
	Sayasone et al 2023 [39]	20120-2017	Lao	Rural	>2	364/257, total 621	Construction of latrines, with minimal material subsidies provided, MDA in dogs. Health education campaigns.	1 year	*S. mekongi*	✓
	Madon et al. 2018 [40]	2015 - 2016	Tanzania	Rural	school children	945/833	Enhanced Development Governance: Health and sanitation education, village clean-up activities, enforcing compliance to sanitation and hygiene standards through fines, introducing income-generating activities, strengthening self-organisational capacity for WASH projects	5 months	*S. mansoni & S. haematobium*	✓

Most observation and intervention studies (69%) used only one diagnostic method to detect schistosomiasis, whereas 30% used two methods or more, and 1% did not specify (Table 2). Duplicate Kato-Katz (i.e., reading two slides from one stool sample) was the most frequently used diagnostic method for detecting *S. mansoni* (55%), whereas more than duplicate Kato-Katz slides for *S. japonicum* (71%). For *S. haematobium*, urine filtration (69%) was most frequently used. Other methods included concentration of eggs followed by microscopy, point-of-care urine circulating cathodic antigen (POC-CCA) assay, assessment of macro-and micro-haematuria, and miracidial hatching. Overall, 11% (11/103) of studies using Kato-Katz did not specify how many slides were analysed per sample.

**Table 2:** Diagnostics for determining the prevalence of schistosomiasis of included observation and intervention studies. % = proportion of diagnostics within each species (column totals = 100%).

Diagnostic method	*S. mansoni*	*S. haematobium*	*S. japonicum*	*S. mekongi*
Unspecified number of Kato-Katz slides	11 (8.7%)			
Single Kato-Katz slide	13 (10.3%)		3 (16.7%)	
Duplicate Kato-Katz slides	53 (42.1%)		3 (16.7%)	1 (25%)
More than duplicate Kato-Katz slides	12 (9.5%)		5 (27.8%)	2 (50%)
Odongo-Aginya stain modification of Kato-Katz	1 (0.8%)			
POC-CCA assay	11 (8.7%)			
Concentration (formal ether, sedimentation or centrifugation) followed by microscopy	19 (15.1%)	24 (26.7%)		
Wet mount	4 (3.2%)			
Urine filtration		44 (48.9%)		
Haematuria (micro and/or macro)		21 (23.3%)		
Miracidium hatching			4 (22.2%)	
Molecular			3 (16.7)	1 (25%)
Questionnaire based on symptoms	1 (0.8%)	1 (1.1%)		
No diagnostic method specified	1 (0.8%)			

## Discussion

This scoping review synthesises the evidence available on the link between WASH exposures and schistosomiasis transmission in humans. Of the 181 studies identified, 160 were observational and 21 were intervention studies. Most observational studies examined domestic, recreational and occupational water contact measures. However, several transmission pathways remain under-examined, including anal cleansing and agricultural use of faecal waste. Although studies on these pathways were screened, they addressed environmental contamination (e.g., snail infection) rather than human infection. While some studies measured WASH facility characteristics (e.g., physical condition, cleanliness, usability for all ages) and their usage, few examined how sustained access and maintenance over time influence infection patterns. This reflects reliance on crude binary (yes/no) access measures, which can misclassify exposure and thereby obscure the relationship between WASH access and quality, and schistosomiasis risk.

Most reviewed studies, both included and excluded, employed a cross-sectional design primarily focused on measuring schistosome infection, either by prevalence or intensity, accompanied by an analysis of WASH-related risk factors. A significant limitation affecting study quality was the frequent absence of a conceptual transmission framework during the study design phase. This oversight led to the exclusion of important exposure pathways such as the near-complete absence of animal reservoir considerations in *S. japonicum* and *S. haematobium* studies, despite the established role of domestic livestock (cattle, water buffalo) in maintaining transmission for these species. This will likely become increasingly important as endemic areas approach elimination and animal reservoirs sustain residual transmission. Other issues included the incorporation of WASH factors unlikely to directly influence infection risk (e.g., consumption of raw vegetables or shoe wearing) and the failure to address all plausible sources of exposure. Together, these issues may obscure the true impact of interventions, either by diluting observed effects or by leaving some transmission pathways unaddressed.

There was considerable variation in the terminology used to describe WASH services. For sanitation, terms ranged from general mentions of latrines, without specification of latrine location or technology, to a specific latrine type and sanitation service. For water, the location of water points, distance to households or quantity of available water was often omitted, which may affect use for bathing or laundry. These gaps likely reflect practical challenges in measuring WASH infrastructure and behaviours, variability in household practices, and the infrequent application of transmission-focused conceptual frameworks (as discussed above). Incorporating both observed and self-reported measures of water quantity and use can strengthen assessments by reducing biases inherent in self-report alone. Standardising infrastructure and service definitions, as proposed by the WHO/UNICEF Joint Monitoring Programme [41], and using observations could improve the consistency and reliability of future study protocols.

A total of 180/580 (31%) of studies were excluded at full-text review because of the failure to differentiate between *Schistosoma* spp., other helminth species, or intestinal protozoa in the analysis, despite often disaggregating prevalence and intensity data by species. This lack of species-specific reporting hinders the ability to accurately interpret the relationship between risk factors and infection. It also highlights that schistosomiasis and STH are still grouped together, despite very different life cycles (e.g., water-contact vs ingestion). This sets up failure in selecting appropriate WASH interventions that attempt to reduce helminth infections. For this reason, Bradley et al. [42] categorised water-related diseases by transmission route. Diseases associated with exposure to surface water and transmission via an aquatic intermediate host (i.e., schistosomiasis) have distinct suggested WASH interventions compared to other WASH-related, such as water-borne or water-washed, diseases. While treatment for helminth infections is often delivered through integrated drug administration campaigns that co-administer praziquantel for schistosomiasis alongside anthelminthic drugs for STH for logistical reasons, WASH interventions should be studied and developed specific to the helminth infection and species.

Despite developments in diagnostic methods and evidence of the low sensitivity of single Kato-Katz in low intensity infections [43,44], 14% of the studies published in the last 20 years still used single Kato-Katz or did not specify how many stool slides were analysed per sample. This likely resulted in under-reporting of infections, which would affect the accuracy of the reports.

### Limitations

Studies may have been missed due to language restrictions, especially for studies on *S. japonicum*, and the choice of databases. We only included peer-review sources, and therefore did not include grey literature and pre-prints, so may have missed some evidence. This review also did not assess the quality or risk of bias of the included studies. Scoping reviews typically do not include a formal assessment of the quality or risk of bias, which means that findings may include evidence of varying quality without differentiation. This review only included studies linking WASH to human infection, not other parts of the lifecycle. Therefore, studies exploring how much WASH activities contribute to environmental contamination (e.g., snail infection, presence of cercariae in water), were excluded. This is the case for studies reporting links between hygienic bathing or anal cleansing and snail infections, not parasitological surveys in humans (e.g., [45,46]).

### Considerations for future research

Further research is needed to better understand the extent to which coverage and use of specific WASH components influence schistosomiasis transmission (Box 1). There are several ways in which study design and reporting could be strengthened to enhance both interpretability and comparability across settings. This would facilitate more robust cross-study comparisons, systematic reviews, and meta-analyses, thereby increasing the overall impact and policy relevance of WASH–schistosomiasis research. Suggested considerations for any future WASH-SCH studies include:

**Box 1. Priorities for future research**

**Table:** 

**Addressing key transmission gaps**	**Prevalence and Intensity:** Analysing risk factors for both prevalence and intensity of infection, to distinguish between the drivers of disease transmission versus the drivers of clinical severity.
	**Animal reservoirs** were largely overlooked, particularly in *S. japonicum* (where bovines are well-established reservoirs) and, to a lesser extent, *S. haematobium*, despite evidence that livestock such as cattle and water buffalo can sustain transmission.
	Key **sanitation-related behaviours**, such as anal cleansing practices and the use of untreated faecal waste in agriculture, were rarely assessed, even though they may contribute to contamination of freshwater bodies with schistosome eggs.
	**Environmental exposure:** Animal presence near households and water treatment practices were seldom examined, despite their potential influence on water contact patterns, cercarial exposure, or contamination of local water sources.
**Transmission informed study design**	**Standardised definitions:** Standardising WASH infrastructure and service definitions, as proposed by the WHO/UNICEF Joint Monitoring Programme [41], could enhance the consistency and reliability of survey protocols.
	Adopting a **conceptual framework**, such as the one outlined in this paper, which focuses on schistosome transmission pathways, could enhance the identification of pre-defined WASH-related factors plausibly associated with infection risk. The framework should be adapted based on species of interest and study context, including environmental, socioeconomic and cultural factors.
	**Species-specific results** should be reported instead of combining helminth or protozoan infections into composite outcomes, as different species respond differently to WASH services, environmental conditions, and behavioural practices. Disaggregated analyses will improve our understanding of transmission pathways and enable the design of more targeted and effective interventions.
**Designing more comprehensive intervention studies**	For intervention studies, additional elements that may improve protocols could incorporate: **Theory of change:** Researchers should develop a clear theory of change outlining how the intervention is expected to interrupt specific transmission pathways. As illustrated in Fig 2, many WASH interventions are not designed to address key transmission routes, such as anal cleansing practices or the application of untreated sludge to fertilise crops. Interventions should therefore be comprehensive and context-specific, and studies should explicitly analyse remaining gaps to prevent misinterpretation of findings. Many studies labelled as “WASH” focus on only one or two components that are unlikely to substantially reduce transmission when implemented in isolation.
	**Setting:** Evaluating only school- or community-level WASH interventions may overlook important exposure domains; studies should instead adopt a more holistic perspective, capturing WASH conditions and water contact behaviours across all settings where people spend their time.
	**Pathway specificity:** Analyses of WASH interventions for schistosomiasis should distinguish between components that directly affect transmission pathways and those with indirect or contextual relevance (e.g., handwashing), as pooling biologically heterogeneous interventions may dilute schistosome-specific effect estimates.
	**Study duration and follow-up:** Intervention studies should account for the persistence of *Schistosoma* eggs in the environment when determining study duration and follow-up periods, ensuring sufficient time to capture ecological and epidemiological changes, including reinfection dynamics. Ideally, studies should measure along the full transmission pathway, including egg presence in the environment (e.g., near latrines) and malacological surveys, alongside traditional epidemiological surveys. This comprehensive approach enables a more complete assessment of the intervention’s impact on infection risk.
	**Intervention fidelity and uptake:** Access to services must be sufficient to meaningfully reduce transmission. Even universal coverage of basic facilities, such as poorly managed latrines or boreholes, may not result in significant reductions in infection. Evaluating intervention fidelity, uptake, and any declines in coverage, usage, or functionality is therefore critical for interpreting results, particularly for sanitation interventions where an individual’s risk is influenced by the practices of others in the community.
	**Data collection:** Where possible, studies should rely on observed, rather than self-reported, data to minimise bias and inaccuracies. Although proxy measures, such as the presence of latrines, can offer some insight into practices, they do not capture actual behaviours or consistent use, which are critical determinants of transmission.

## Conclusion

This scoping review highlights important gaps and opportunities in the evidence base linking WASH and schistosomiasis transmission. Empirical support is strongest for water contact and sanitation access, where a substantial body of observational research exists. However, critical transmission pathways, such as anal cleansing, agricultural use of faecal waste, and animal reservoirs in *S. haematobium*, remain unexamined. The frequent absence of conceptual frameworks of transmission has led to the inclusion of irrelevant WASH variables and the omission of key factors. The lack of standardised WASH definitions, inconsistent species-specific reporting, and underuse of sensitive diagnostics further limit comparability across studies and the strength of current evidence.

To improve the design and implementation of future WASH research for schistosomiasis, we recommend adopting standardised protocols and definitions, species-specific reporting, and conceptual frameworks grounded in transmission dynamics. Intervention studies would benefit from clear theories of change, longer follow-up periods, assessment of intervention fidelity and uptake, and attention to both individual- and community-level exposures. Addressing these research gaps and improving methodological rigor will be essential to optimise WASH interventions as part of integrated schistosomiasis control and elimination strategies.

## Supporting information

S1 File PRISMA Checklist. (DOCX)

S1 Table Search terms. (DOCX)

S2 Table Included observational studies with references. (DOCX)

S3 Table Additional data extracted from observational studies. (XLSX)
