# Supplementary material for: Water, sanitation, and hygiene in schistosomiasis control: A scoping review of evidence gaps across transmission pathways
Source: PLOS Glob Public Health. 2026 Jul 30;6(7):e0006916. doi: 10.1371/journal.pgph.0006916 (PMC13423035; doi:10.1371/journal.pgph.0006916)
Supplement: S1 Table — (DOCX) [file pgph.0006916.s002.docx]

***S2 Table: Search terms***

| **Heading 1** | (Schistosom* or Mansoni or Haematobium or Japonicum or Mekongi or Bilharzia).ti,ab. or Schistosomiasis/  AND |
| --- | --- |
| **Heading 2** | (disinfect* or sanita* or water treatment or hygiene or toilet* or sewage or borehole* or standpipe or rainwater or latrine* or pit or “open defecation” or urination or “fecal sludge” or “faecal sludge” or slurry or effluent or shower* or bath* or laundry or “piped water” or pipe or “surface water” or irrigation or “water supply” or chlorination or “treated water” or “water filter” or “water filtration” or handwashing or “hand washing” or hand-washing or soap or “swimming pool” or “recreational pool”).ti,ab. or disinfection/ or sanitation/ or water treatment/ or hygiene/  AND |
| **Heading 3** | (incidence or prevalen* or epidemi* or infect* or surveill* or improve* or presence*).ti,ab |
