# Supplementary material for: Water, sanitation, and hygiene in schistosomiasis control: A scoping review of evidence gaps across transmission pathways
Source: PLOS Glob Public Health. 2026 Jul 30;6(7):e0006916. doi: 10.1371/journal.pgph.0006916 (PMC13423035; doi:10.1371/journal.pgph.0006916)
Supplement: S2 Table — (DOCX) [file pgph.0006916.s003.docx]

***S3 Table: Description of included observational studies. NA= not available, SAC = school-aged children***

| **Author(s)** | **Publication year** | **Year of study** | **Country** | **Region** | **Setting** | **Number of participants** | **Age of participants** | **Schistosoma species** |
| --- | --- | --- | --- | --- | --- | --- | --- | --- |
| Abera et al (1) | 2025 | 2022 | Ethiopia | Mizan-Aman | Both | 615 | 5-14 | *Mansoni* |
| Abie et al (2) | 2023 | 2019 | Ethiopia | Northwest | Rural | 588 | 6-14 | *Mansoni* |
| Abou-Zeid et al (3) | 2012 | 2009 | Sudan | South Kordofan | Rural & urban | 1,826 | 18+ | *Haematobium* |
| Al-Murisi et al (4) | 2022 | 2019 | Yemen | An-Nadirah District | Rural | 417 | 6 to 15 | *Mansoni* |
| Al-Shibani et al (5) | 2007 | NA | Yemen | Taiz | Rural | 152 | SAC | *Mansoni* |
| Al-Waleedi et al (6) | 2013 | 2011 | Yemen | Sana’a. | Not specified | 696 | 10 to 16 | *Haematobium* |
| Alembrhan et al (7) | 2013 | 2010 | Ethiopia | Mekelle | Sub-urban | 457 | SAC | *Mansoni* |
| Alharazi et al (8) | 2022 | 2019 | Yemen | Taiz | Rural | 478 | 6-15 | *Mansoni* |
| Alidou et al (9) | 2025 | 2022 | Togo | Plateau | Rural | 6,400 | 5-14 | Haematobium |
| Aliyi et al (10) | 2024 | 2019 | Ethiopia | East | Rural | 419 | 6-19 | *Mansoni* |
| Amorim et al (11) | 1997 | NA | Brazil | Minas Gerais | Both | 1,067 | ≥ 10 | *Mansoni* |
| Amuta et al (12) | 2014 | 2012 | Nigeria | Guma | Rural | 300 | 1 to 15 | *Haematobium* |
| Anaba (13) | 2025 | 2023 | Ghana | Ashanti | Rural | 333 | 6-18 | Haematobium |
| Andargie et al (14) | 2018 | 2015 | Ethiopia | Tach-Armachiho district | Rural | 228 | 0 to > 40 | *Mansoni* |
| Ansha et al (15) | 2020 | 2018 | Ethiopia | Wondo district | Not specified | 298 | 5 to 19 | *Mansoni* |
| Aribodor et al (16) | 2024 | 2023 | Nigeria | Anambra | Rural | 470 | 10 to 19 | *Haematobium* |
| Atalabi et al (17) | 2018 | 2016 | Nigeria | Katsina State | Rural | 484 | 6 to 15 | *Haematobium* |
| Atalabi et al (18) | 2016 | 2015 | Nigeria | Katsina state | Rural | 718 | 10 to 23 | *Haematobium* |
| Auta et al (19) | 2023 | 2021 | Nigeria | Kano state | Not specified | 400 | 5-10 | Haematobium |
| Awoke et al (20) | 2013 | 2012 | Ethiopia | Amibera | Rural | 832 | 5 to 24 | *Haematobium* |
| Ayeh-Kumi et al (21) | 2015 | 2009 | Ghana | Ashanti | Rural | 100 | 4 to 17 | *Haematobium* |
| Bakr et al (22) | 2009 | 2003 | Senegal | Thiago | Peri-urban | 991 | ≥ 5 | *Mansoni* |
| Balen et al (23) | 2011 | 2006 | China | Hunan | Peri-urban | 1,298 | All ages | *Japonicum* |
| Balogun et al (24) | 2022 | 2020 | Nigeria | Jigawa State | Rural | 279 | 5 to 16 | *Haematobium* |
| Barreto (25) | 1991 | 1984 | Brazil | Santo Antonio | Urban | 1,494 | 12 to 15 | *Mansoni* |
| Bartlett et al (26) | 2022 | 2014 | Angola | Huambo, Uige, Zaire | Rural | 17,093 | 9 to 14 | *Mansoni & Haematobium* |
| Bekana et al (27) | 2021 | 2018 | Ethiopia | Amhara | Rural | 798 | 6 to 15 | *Mansoni* |
| Binga et al (28) | 2022 | 2021 | Nigeria | Taraba | Both | 384 | All | haematobium |
| Bishop et al (29) | 2023 |  | Nigeria | Kaduna | Rural | 600 | 10-19 | Haematobium |
| Bolaji et al (30) | 2015 | 2013 | Nigeria | Kwara State | Rural | 150 | 9- >16 | *Haematobium* |
| Byagamy et al (31) | 2025 | 2023 | Uganda | Lango | Rural | 802 | 5-16 | *Mansoni* |
| Campbell et al (32) | 2017 | 2015 | Cameroon | Barombi Mbo and Barombi Kotto lakes | Rural | 338 | 1 to 83 | *Haematobium* |
| Carlton et al (33) | 2009 | 2007 - 2010 | China | Sichuan | Rural | 2,005 | ≥ 6 | *Japonicum* |
| Coura-Filho (34) | 1994 | NA | Brazil | All | Both | 4,500 | 6 to 34 | *Mansoni* |
| Coura-Filho et al (35) | 1994 | 1991 | Brazil | Peri-Peri | Urban | 337 | >1 | *Mansoni* |
| Coura-Filho et al (36) | 1995 | 1980 | Brazil | Ravena | Urban | 998 | 10 to 19 | *Mansoni* |
| Cundill et al (37) | 2011 | 2004-2006 | Brazil | Americaninhas | Both | 598 | ≥5 | *Mansoni* |
| da Silva (38) | 1997 | NA | Brazil | Cururupu | Rural | 294 | All ages | *Mansoni* |
| Dalton and Pole (39) | 1978 | NA | Ghana | Lake Volta | Rural | 132 | All ages | *Haematobium* |
| Damen et al (40) | 2006 | NA | Nigeria | Kaduna | Rural | 306 | 10 to 25 | *Mansoni & Haematobium* |
| Damtie et al (41) | 2021 | 2019-2020 | Ethiopia | Libokemkem District | Rural | 277 | 18-45 | *Mansoni* |
| Dassah et al (42) | 2023 | 2022 | Ghana | Upper East Region | Rural | 326 | 9-17 | *Mansoni* |
| Dawet et al (43) | 2012 | NA | Nigeria | Plateau | Rural & urban | 242 | All ages | *Haematobium* |
| de Lima e Costa (44) | 1987 | 1981 | Brazil | Comercinho | NA | 1,064 | ≥2 | *Mansoni* |
| de Lima e Costa (45) | 1991 | NA | Brazil | Divino | NA | 506 | ≥2 | *Mansoni* |
| de Lima e Costa (46) | 1994 | NA | Brazil | Minas Gerais | NA | 1,162 | ≥2 | *Mansoni* |
| Enk et al (47) | 2010 | NA | Brazil | Minas Gerais | Both | 1,061 | All ages | *Mansoni* |
| Erismann et al (48) | 2016 | 2015 | Burkina | Plateau central | Rural | 385 | 8 to 14 | *Haematobium* |
| Exum et al (49) | 2019 | 2016 - 2017 | Uganda | Whole country | Rural & urban | 9,097 | 2 to 50+ | *Mansoni* |
| Farooq et al (50) | 1966 | 1962-63 | Egypt | Multiple | Rural & urban | 23,572 | All ages | *Mansoni & Haematobium* |
| Fentahun et al (51) | 2021 | 2021 | Ethiopa | Lake Tana | Rural | 388 | All ages | *Mansoni* |
| Fentie et al (52) | 2013 | 2007-2008 | Ethiopia | Lake Tana Basin | Rural | 520 | 6 to 15 | *Mansoni* |
| Fetene et al (53) | 2021 | 2017 | Ethiopia | Bahir Dar Zuria District | Rural | 394 | 6 to 14 | *Mansoni* |
| Firmo et al (54) | 1996 | 91 to 92 | Brazil | Minas Gerais | Urban | 916 | All ages | *Mansoni* |
| Fuerst (55) | 2013 | 2010 | Cote d'Ivoire | South-centre | Rural | 195 | All ages | *Mansoni* |
| Ghazy et al (56) | 2022 | 2019 | Egypt | Kafr El-Sheikh | Rural | 861 | 6-15 | *Mansoni* |
| Gichuki et al (57) | 2019 | 2017 | Kenya | Mwea | Rural | 905 | 17 to 95 | *Mansoni* |
| Gomes et al (58) | 2025 | 2014 - 2022 | Brazil | Minas Gerais | Rural | 265 | 6-69 | *Mansoni* |
| Guimarães et al (59) | 1985 | NA | Brazil | Tuparence | Not specified | 745 | All ages | *Mansoni* |
| Guimarães et al (60) | 1985 | 1983 | Brazil | Ilha | Rural & urban | 167 | SAC | *Mansoni* |
| Guo and Jiang (61) | 2004 | 2002 | China | Jiangxi | Rural | 336 | 10 to 65 | *Japonicum* |
| Hailegebriel et al (62) | 2021 | 2021 | Ethiopia | Lake Tana | Rural | 681 | 5 to 22 | *Mansoni* |
| Hailu et al (63) | 2020 | 2016 | Ethiopia | Jawe district | Rural | 333 | 6 to 14 | *Mansoni* |
| Hailu et al (64) | 2018 | 2017 | Ethiopia | Bahir Dar | Rural & urban | 409 | 7 to 14 | *Mansoni* |
| Hilali et al (65) | 1995 | 1992-1994 | Sudan | Managil | Rural | 1,450 | All ages | *Mansoni* |
| Houmsou et al (66) | 2010 | 2008-2009 | Nigeria | Benue state | NA | 750 | 3 to 70 | *Haematobium* |
| Ibrahim et al (67) | 2018 | 2016 | Ethiopia | Ejaji | Rural & urban | 240 | 6 to 19 | *Mansoni* |
| Ito et al (68) | 2025 | 2020 to 2022 | Nigeria | Southern | Rural | 7,219 | 10-50 | Haematobium |
| Jeza et al (69) | 2022 | 2018 | Kenya | Kwale county | Rural | 534 | 15 to 50 | *Haematobium* |
| Joof et al (70) | 2021 | 2015 | Gambia | all | Rural | 2,018 | 7 to 14 | *Haematobium* |
| Kabatereine et al (71) | 2011 | 2009 to 10 | Uganda | Lake Victoria | Rural & urban | 1,784 | 10 to 14 | *Mansoni* |
| Kabuyaya et al (72) | 2017 | 2015 | South Africa | uMkhanyakude district | Rural | 320 | 10 to 15 | *Haematobium* |
| Kajembe (73) | 2022 | NA | Tanzania | Mkuranga | Rural | 396 | 6-16 | Haematobium |
| Kim et al (74) | 2024 | 2022 | Uganda | Mayuge | Rural | 1,617 | 7-21 | *Mansoni* |
| Knopp et al (75) | 2013 | 2006 - 2009 | Tanzania (Zanzibar) | Unguja island | Rural | 879 | 0 to 100 | *Haematobium* |
| Kulinkina et al (76) | 2019 | 2015 | Ghana | Eastern region | Rural | 897 | SAC | *Haematobium* |
| Kumagai et al (77) | 2022 | 2016 | Laos | Champasak | Rural | 272 | 5-60 | *Mekongi* |
| Lakew et al (78) | 2015 | 2010 | Ethiopia | Jimma town | Peri-urban | 116 | All ages | *Mansoni* |
| M'Bra et al (79) | 2018 | 2015 | Cote d'Ivoire | Korhogo | Urban | 2,341 | 5 to 15 | *Mansoni* |
| Mahmud et al (80) | 2013 | NA | Ethiopia | North | Rural & urban | 600 | 6 to 15 | *Mansoni* |
| Malibiche (81) | 2023 | 2022 | Tanzania | Nachingwea | Rural | 483 | 7-14 | *Haematobium* |
| Marcal Junior et al (82) | 1993 | 1987 | Brazil | Pedro Toledo | Rural & urban | 192 | All ages | *Mansoni* |
| Masaku et al (83) | 2020 | 2018 | Kenya | Busia County | Rural | 653 | 3 to 7 | *Mansoni* |
| Maseke et al (84) | 2022 | 2021 | Tanzania | Itilima | Rural | 433 | 13-21 | *Haematobium* |
| Massara et al (85) | 2004 | 2001-2003 | Brazil | Belo Horizonte | Both | 1,186 | All ages | *Mansoni* |
| Mathewos et al (86) | 2014 | 2012 | Ethiopia | North Gondar | Rural | 261 | 5 to 15 | *Mansoni* |
| Matthys et al (87) | 2007 | NA | Cote d'Ivoire | Man | Urban | 716 | All ages | *Mansoni* |
| Mohammed et al (88) | 2023 | 2021 | Ethiopia | Kurmuk | Rural | 403 | 5 and above | *Haematobium* |
| Mota and Sleigh(89) | 1987 | NA | Brazil | Northeast | Rural | 141 | All ages | *Mansoni* |
| Moza et al (90) | 1998 | 1994 | Brazil | Pernambuco | Rural | 93 | ≥ 2 | *Mansoni* |
| Munisi et al (91) | 2016 | NA | Tanzania | Rorya District | Rural | 513 | 6 to 16 | *Mansoni* |
| Murenjekwa et al (92) | 2021 | 2012 - 2015 | Zimbabwe | Midland’s province | Rural | 4,437 | NA, median 25.5 years | *Haematobium* |
| Musuva et al (93) | 2021 | NA | Kenya | Nyanza Province | Rural | 1,200 | 9-12 and adults | *Mansoni* |
| Mutsaka-Makuvaza et al (94) | 2019 | NA | Zimbabwe | Mashonaland Central Province | Rural | 860 | 0-5 and 15-50 | *Haematobium* |
| N'Zi et al (95) | 2021 | 2016 -2018 | Cote d'Ivoire | Tonkpi region | Rural | 350 | 1 to 6 | *Mansoni* |
| Ndassa et al (96) | 2007 | 2001 -2002 | Cameroon | Upper Benue Valley | Rural | 766 | All | *Haematobium* |
| Ndokeji et al (97) | 2016 | NA | Tanzania | Ilemela District | Rural | 454 | 4 to 14 | *Mansoni* |
| Ndyomugyenyi and Minjas (98) | 2001 | NA | Tanzania | Dar Es Salaam | Urban | 483 | 5 to 19 | *Haematobium* |
| Ngui et al (99) | 2024 | 2022 | Kenya | Kwale | Rural | 422 | 15-50 | *Haematobium* |
| Nigo et al (100) | 2021 | 2016- 2017 | DRC | Ituri province | Rural & urban | 2,838 | >1 | *Mansoni* |
| Njambi et al (101) | 2020 | NA | Kenya | Kirinyaga County | Rural | 180 | 8 to 14 | *Mansoni* |
| Noman et al (102) | 2012 | 2006 | Yemen | Taiz | Not specified | 210 | 0-17 | *Mansoni & Haematobium* |
| Nworie et al (103) | 2012 |  | Nigeria | Ebonyi state | Rural | 500 | 5 to 15 | *Haematobium* |
| Nyirenda et al (104) | 2022 | NA | Zambia | Kafue | Rural | 322 | 6-20 | *Haematobium* |
| Obadiah et al (105) | 2018 | 2016 | Nigeria | Benue State | Rural | 700 | ≤5 to ≥ 20 | *Haematobium* |
| Ojeleye et al (106) | 2024 | 2021 to 2022 | Nigeria | Kaduna | Rural | 200 | 8-17 | *Haematobium* |
| Okoyo et al (107) | 2020 | 2017 | Kenya | 6 regions / national | Rural | 9,801 | 1 to 21 | *Mansoni* |
| Okoyo et al (108) | 2021 | 2017 | Kenya | 6 regions / national | Rural | 199 schools | Class 2-6 | *Mansoni* |
| Omondi et al (109) | 2021 | 2018 | Kenya | Siaya and Homa Bay counties | Rural | 600 | 18 to 90 | *Mansoni* |
| Onyekwere et al (110) | 2022 | 2019 | Nigeria | 7 states in south Nigeria | Rural | 5,514 | 5 to 14 | *Haematobium* |
| Opara et al (111) | 2021 | 2017-2018 | Nigeria | Cross River State | Rural | 630 | 5 to 16 | *Haematobium* |
| Opoku-Kwabi (112) | 2024 | 2023 | Ghana | Adisadel | Peri-urban | 246 | 4-19 | Both |
| Paller et al (113) | 2024 | 2019-2021 | Philippines | Mindanao | Not specified | 386 | 10-60 | *Japonicum* |
| Palmeira et al (114) | 2010 | NA | Brazil | Alagos | Urban | 329 | 7 to 15+ | *Mansoni* |
| Phillips et al (115) | 2023 | 2018-2019 | Ethiopia | Wolayita | Rural | 6,637 | 0->36 | *Mansoni* and *Haematobium* |
| Phiri et al (116) | 2016 | 2005 | Malawi | Chikwawa district | Rural | 1,642 | >1 | *Haematobium* |
| Reitzug et al (117) | 2023 | 2022 | Uganda | East and West | Rural | 2,867 | 5-90 | *Mansoni* |
| Reuben et al (118) | 2013 | NA | Nigeria | Lafia | Urban & sub-urban | 160 | SAC | *Haematobium* |
| Risikat et al (119) | 2012 | NA | Nigeria | Ijoun | Rural | 268 | 5 to 16 | *Haematobium* |
| Rodrigues et al (120) | 1995 | NA | Brazil | Itinga | Not specified | 324 | All ages | *Mansoni* |
| Rogers et al (121) | 2024 | 2020 | Uganda | Buyende | Rural | 348 | 5-15 | *Mansoni* |
| Rollemberg et al (122) | 2015 | 2008 - 2010 | Brazil | Sergipe | Rural | 500 | 9 to 73 | *Mansoni* |
| Ross et al (123) | 2017 | 2012 | Philippines | North Samar | Rural | 6,976 | All | *Japonicum* |
| Ruganuza et al (124) | 2015 | NA | Tanzania | Ukerewe Island | Rural | 400 | 1 to 6 | *Mansoni* |
| Rujeni et al (125) | 2022 | NA | Rwanda | Nationwide | Rural | 4,675 | 7 - 68 months | *Mansoni* |
| Sady et al (126) | 2013 | NA | Yemen | West | Rural | 400 | <15 | *Mansoni* |
| Salawu et al (127) | 2016 | 2010 - 2011 | Nigeria | Ogun State | Rural | 237 | 15 to 30+ | *Mansoni & Haematobium* |
| Samweli et al (128) | 2023 | 2022 | Tanzania | Shinyanga | Rural | 620 | 11-20 | *Mansoni* |
| Schmidlin et al (129) | 2013 | 2011 | Cote d'Ivoire | Taabo, South-central | Rural | 1,894 | All ages | *Haematobium* |
| Sekre et al (130) | 2024 | 2022 | Cote d’Ivoire | West | Rural | 1,602 | 5-14 | *Mansoni* |
| Shabani et al (131) | 2022 | 2020 | Tanzania | Muleba | Rural | 328 | 18-55 | *Mansoni* |
| Silva et al (132) | 2020 | 2015 | Brazil | Bahia | Urban | 1,654 | All ages | *Mansoni* |
| Sitotaw et al (133) | 2020 | 2018 - 2019 | Ethiopia | Sasiga District | Urban | 383 | < 5 to 18 | *Mansoni* |
| Soares et al (134) | 1995 | NA | Brazil | Paracambi | Peri-urban | 1196 | All ages | *Mansoni* |
| Sousa-Figueiredo et al (135) | 2015 | NA | Namibia | northern | Rural | 17,896 | 3 to 19 | *Mansoni & Haematobium* |
| Stephenson et al (136) | 1986 | NA | Kenya | Kwale | Rural | 105 | NA | *Haematobium* |
| Stothard et al (137) | 2002 | 2001 | Zanzibar, Tanzania | Unguja | NA | 400 | 8 to 19 | *Haematobium* |
| Sudat, et al (138) | 2010 | 2000 | China | Xichang | Rural | 1,011 | All ages | *Japonicum* |
| Surakat et al (139) | 2020 | 2019 | Nigeria | Osun state | Semi-urban & rural | 243 | 5 - 16 | *Haematobium* |
| Tadege et al (140) | 2017 | 2015 | Ethiopia | Lake Hawassa | Rural | 374 | 5 to 15 | *Mansoni* |
| Tadesse et al (141) | 2013 | 2012 | Ethiopia | Raya Alamata district | Rural | 500 | 6 to 19 | *Mansoni* |
| Tanser et al (142) | 2018 | 2015 | South Africa | uMkhanyakude district | Rural | 2,105 | 9 to 14+ | *Haematobium* |
| Tazebew et al (143) | 2022 | 2018 | Ethiopia | Guangua District | Rural | 404 | 5 to 19 | *Mansoni* |
| Tiruneh et al (144) | 2020 | 2019 | Ethiopia | Abeshge district | Rural | 389 | 5 to 15 | *Mansoni* |
| Tolera et al (145) | 2024 | 2024 | Ethiopia | west Shoa zone | Both | 338 | 7-18 | *Mansoni* |
| Tupps et al (146) | 2022 | 2018 | Sierra Leone | Bo and Kenema districts | Both | 2.692 | 1 to 59 | *Mansoni* |
| Umar (147) | 2000 | NA | Nigeria | Bakalori | Rural | 240 | 9 to 16 | *Haematobium* |
| Usman & Babeker (148) | 2017 | 2016 | Nigeria | Bauchi State | Rural | 2,000 | 4 to 41+ | *Haematobium* |
| Usman (149) | 2020 | 2016 | Nigeria | Bauchi Central Senatorial Zone | Rural | 600 | 4 to 41+ | *Haematobium* |
| Vonghachack et al (150) | 2017 | 2011-2012 | Lao PDR | Mekong Islands | Rural | 994 | 2 to 88 | *Mekongi* |
| Wanjala et al (151) | 2013 | 2006 - 2008 | Kenya | Western | Rural | 972 | 5 to 14 | *Mansoni* |
| Watts and Katsha (152) | 1995 | 1991 | Egypt | Nile Delta | Rural | 967 | All ages | *Mansoni* |
| Wepnje et al (153) | 2019 | 2016 - 2018 | Cameroon | Munyenge | Rural | 368 | 15 to 42 | *Haematobium* |
| Wubet et al (154) | 2020 | 2018 - 2019 | Ethiopia | Northwest | Rural | 362 | 7 to 15+ | *Mansoni* |
| Ximenes et al (155) | 2003 | NA | Brazil | São Lourenço da Mata | Urban | 1,674 | 10 to 25 | *Mansoni* |
| Yahaya et al (156) | 2024 | 2023 | Nigeria | Zamfara state | Not specified | 551 | 5-20 | Haematobium |
| Yang et al (157) | 2009 | NA | China | Hunan | Not specified | 10, 108 | ≥6 | *Japonicum* |
| Yangaza et al (158) | 2022 | 2020 | Tanzania | Dar es Salaam | urban | 250 | 7-18 | Haematobium |
| Yusuf et al (159) | 2025 | 2024 | Nigeria | Bauchi | Both | 384 | 6-15 | Haematobium |
| Zeleke et al (160) | 2020 | 2018 | Ethiopia | Maksegnit, Debark, Sanja, Chuahit districts | Rural | 786 | 7 to 16 | *Mansoni* |

1. Abera M, Belay T, Emana D, Mekonnen Z. Assessing soil-transmitted helminths and Schistosoma mansoni infections using parasitological indicators after seven years of preventive chemotherapy among school-age children in Mizan-Aman town. PLoS Negl Trop Dis. 2025;19(5):e0013058.

2. Abie A, Hailu T, Alemu G, Nibret E, Amor A, Munshea A. Prevalence of Soil-Transmitted Helminths and Schistosoma mansoni among Schoolchildren across Altitudinal Gradients in Amhara National Regional State, Ethiopia. Am J Trop Med Hyg. 2023;109(3):667-75.

3. Abou-Zeid AH, Abkar TA, Mohamed RO. Schistosomiasis and soil-transmitted helminths among an adult population in a war affected area, Southern Kordofan state, Sudan. Parasit Vectors. 2012;5:133.

4. Al-Murisi WMS, Al-Mekhlafi AM, Mahdy MAK, Al-Haidari SA, Annuzaili DA, Thabit AAQ. Schistosoma mansoni and soil-transmitted helminths among schoolchildren in An-Nadirah District, Ibb Governorate, Yemen after a decade of preventive chemotherapy. PLoS ONE. 2022;17(8):e0273503.

5. Al-Shibani LA, El-Heggiagi MB, Burshan NM, Bassiouny HK. Development of schistosomal school-based health education model for Yemeni schoolchildren. J Egypt Soc Parasitol. 2007;37(2):649-58.

6. Al-Waleedi AA, El-Nimr NA, Hasab AA, Bassiouny HK, Al-Shibani LA. Urinary schistosomiasis among schoolchildren in Yemen: prevalence, risk factors, and the effect of a chemotherapeutic intervention. J Egypt Public Health Assoc. 2013;88(3):130-6.

7. Assefa A, Dejenie T, Tomass Z. Infection prevalence of Schistosoma mansoni and associated risk factors among schoolchildren in suburbs of Mekelle city, Tigray, Northern Ethiopia. Momona Ethiopian Journal of Science. 2013;5(1):174-88.

8. Alharazi T. Intestinal Parasitic Infection Among Rural Schoolchildren in Taiz, Yemen: School-based Assessment of The Prevalence and Associated Risk Factors. Helminthologia. 2022;59(3):233-45.

9. Alidou S, Kamassa HE, Lack F, Ataba E, Fleming FM, Sossou E, et al. Risk factors associated with urogenital schistosomiasis: a multilevel assessment approach using an Oversampling Schistosomiasis Survey (SOS) community-based, Plateaux region, Togo 2022. BMJ Public Health. 2025;3(1):e001304.

10. Aliyi H, Ahmed M, Gobena T, Alemu BM, Abdi Adem H, Aliyi Usso A. Prevalence and factors associated with Schistosoma mansoni infection among primary school children in Kersa District, Eastern Ethiopia. Peerj. 2024;12:e17439.

11. Amorim MN, Rabello A, Contreras RL, Katz N. Epidemiological characteristics of Schistosoma mansoni infection in rural and urban endemic areas of Minas Gerais, Brazil. Mem Inst Oswaldo Cruz. 1997;92(5):577-80.

12. Amuta EU, Houmsou RS. Prevalence, intensity of infection and risk factors of urinary schistosomiasis in pre-school and school aged children in Guma Local Government Area, Nigeria. Asian Pacific Journal of Tropical Medicine. 2014;7(1):34-9.

13. Anaba E, Tekpertey R, Boakye AE. Examining the Awareness Level, Prevalence and the Risk Factors that Reinforce the Schistosoma Lifecycle and its Transmission Among Children in the Bosomtwe District, Ghana. Ssrn. 2025;10.

14. Andargie AA, Abera AS. Determinants of Schistosoma mansoni in Sanja health center, north West Ethiopia. BMC Public Health. 2018;18(1):620.

15. Ansha MG, Kuti KA, Girma E. Prevalence of Intestinal Schistosomiasis and Associated Factors among School Children in Wondo District, Ethiopia. Journal of Tropical Medicine. 2020;2020.

16. Aribodor OB, Azugo NO, Jacob EC, Ngenegbo UC, Onwusulu ND, Obika I, et al. Assessing urogenital schistosomiasis and female genital schistosomiasis (FGS) among adolescents in Anaocha, Anambra State, Nigeria: implications for ongoing control efforts. BMC Public Health. 2024;24(1):952.

17. Atalabi TE, Adoh SD, Eze KM. The current epidemiological status of urogenital schistosomiasis among primary school pupils in Katsina State, Nigeria: An imperative for a scale up of water and sanitation initiative and mass administration of medicines with Praziquantel. PLoS Negl Trop Dis. 2018;12(7):e0006636.

18. Atalabi TE, Lawal U, Ipinlaye SJ. Prevalence and intensity of genito-urinary schistosomiasis and associated risk factors among junior high school students in two local government areas around Zobe Dam in Katsina State, Nigeria. Parasit Vectors. 2016;9(1):388.

19. Auta T, Gbaden SK, Atalabi TE. Endemicity of urogenital schistosomiasis and its associated risk factors among children in danbatta, northwestern Nigeria. Biomedicine and Chemical Sciences. 2023;2(3):208-16.

20. Awoke W, Beyene MB, Tarekegn M. Prevalence of schistosomiasis and associated factors among students attending at elementary schools in Amibera District, Ethiopia. Open Journal of Preventive Medicine. 2013;03:199-204.

21. Ayeh-Kumi PF, Obeng-Nkrumah N, Baidoo D, Teye J, Asmah RH. High levels of urinary schistosomiasis among children in Bunuso, a rural community in Ghana: an urgent call for increased surveillance and control programs. Journal of Parasitic Diseases. 2015;39:613-23.

22. Bakr I, Arafa N, Ahmed M, Mostafa M, Mohamed M. Prevalence of intestinal parasitosis in a rural population in Egypt, and its relation to socio-demographic characteristics. Journal of the Egyptian Society of Parasitology. 2009;39:371-81.

23. Balen J, Raso G, Li Y-S, Zhao Z-Y, Yuan L-P, Williams GM, et al. Risk factors for helminth infections in a rural and a peri-urban setting of the Dongting Lake area, People’s Republic of China. International Journal for Parasitology. 2011;41(11):1165-73.

24. Balogun JB, Adewale B, Balogun SU, Lawan A, Haladu IS, Dogara MM, et al. Prevalence and Associated Risk Factors of Urinary Schistosomiasis among Primary School Pupils in the Jidawa and Zobiya Communities of Jigawa State, Nigeria. Annals of Global Health. 2022;88(1).

25. Barreto ML. Geographical and socioeconomic factors relating to the distribution of Schistosoma mansoni infection in an urban area of north-east Brazil. Bull World Health Organ. 1991;69(1):93-102.

26. Bartlett AW, Sousa-Figueiredo JC, van Goor RC, Monaghan P, Lancaster W, Mugizi R, et al. Burden and factors associated with schistosomiasis and soil-transmitted helminth infections among school-age children in Huambo, Uige and Zaire provinces, Angola. Infectious Diseases of Poverty. 2022;11(1):73.

27. Bekana T, Berhe N, Eguale T, Aemero M, Medhin G, Tulu B, et al. Prevalence and factors associated with intestinal schistosomiasis and human fascioliasis among school children in Amhara Regional State, Ethiopia. Tropical Medicine and Health. 2021;49(1).

28. Binga WE, Houmsou RS, Garba LC, Amuta EU, Santaya KL. Use of rivers' water, inadequate hygiene, and sanitation as exposure of internally displaced persons (IDPs) to urogenital schistosomiasis and soil-transmitted helminthiasis in Jalingo Local Government Area (LGA), Taraba State, Nigeria. Journal of Water, Sanitation and Hygiene for Development. 2022;12(11):792-802.

29. Bishop HG, Inabo HI, Ella EE, Bello M. Urinary schistosomiasis: risk factors and symptoms among school adolescents in Kaduna State, Nigeria. EUREKA Life Sciences. 2023;15(2):56-62.

30. Bolaji OS, Elkanah FA, Ojo JA, Ojurongbe O, Adeyeba OA. Prevalence and intensity of Schistosoma haematobium among school children in Ajase-Ipo, Kwara State, Nigeria. Asian Journal of Biomedical and Pharmaceutical Sciences. 2015;5(43):6-11.

31. Byagamy JP, Opiro R, Nyafwono M, Malinga GM, Echodu R, Odongo-Aginya EI. Prevalence, intensity, and risk factors of Schistosomiasis and Intestinal Parasitic Infections among primary school children in northern Uganda: Implications for public health interventions. medRxiv. 2025;14.

32. Campbell SJ, Stothard JR, O'Halloran F, Sankey D, Durant T, Ombede DE, et al. Urogenital schistosomiasis and soil transmitted helminthiasis (STH) in Cameroon: an epidemiological update at Barombi Mbo and Barombi Kotto crater lakes assessing prospects for intensified control interventions. Infectious Diseases of Poverty. 2017;6(49).

33. Carlton EJ, Liu Y, Zhong B, Hubbard A, Spear RC. Associations between Schistosomiasis and the Use of Human Waste as an Agricultural Fertilizer in China. PLOS Neglected Tropical Diseases. 2015;9(1):e0003444.

34. Coura-Filho P. [The use of risk factor determination for schistosomiasis in endemic areas in Brazil]. Cadernos de saude publica. 1994;10 4:464-72.

35. Coura-Filho P, Rocha RS, Farah MW, Silva GCd, Katz N. Identification of factors and groups at risk of infection with Schistosoma mansoni: a strategy for the implementation of control measures? Revista do Instituto de Medicina Tropical de São Paulo. 1994;36:245-53.

36. Coura-Filho P, Farah MW, Rezende DF, Lamartine SS, Carvalho OS, Katz N. [Environmental and social determinants in schistosomiasis mansoni in Ravena, Minas Gerais, Brazil]. Cad Saude Publica. 1995;11(2):254-65.

37. Cundill B, Alexander N, Bethony JM, Diemert D, Pullan RL, Brooker S. Rates and intensity of re-infection with human helminths after treatment and the influence of individual, household, and environmental factors in a Brazilian community. Parasitology. 2011;138(11):1406-16.

38. Silva AAMd, Cutrim RNM, Alves MTSSdB, Coimbra LC, Tonial SR, Borges DP. Water-contact patterns and risk factors for Schistosoma mansoni infection in a rural village of northeast Brazil. Revista do Instituto de Medicina Tropical de Sao Paulo. 1997;39:91-6.

39. Dalton P, Pole D. Water-contact patterns in relation to Schistosoma haematobium infection. Bulletin of the World Health Organization. 1978;56(3):417.

40. Damen J, Banwat E, Egah D, Shabi M. Schistosomiasis among students in a local government area of Kaduna State in Northern Nigeria. Highland Medical Research Journal. 2006;4(1):60-9.

41. Damtie D, Sitotaw B, Menkir S, Kerisew B, Hussien K. Human Intestinal Parasitic Infections: Prevalence and Associated Risk Factors among Elementary School Children in Merawi Town, Northwest Ethiopia. Journal of Parasitology Research. 2021;2021.

42. Dassah SD, Nyaah KE, Senoo DKJ, Ziem JB, Aniweh Y, Amenga-Etego L, et al. Co-infection of Plasmodium falciparum and Schistosoma mansoni is associated with anaemia. Malar J. 2023;22(1):272.

43. Dawet A. Prevalence and intensity of Schistosoma haematobium among residents of Gwong and Kabong in Jos north local government area, Plateau State, Nigeria. International Journal of Biological and Chemical Sciences. 2012;6(4):1557-65.

44. e Costa ML, Magalhaes M, Rocha R, Antunes C, Katz N. Water-contact patterns and socioeconomic variables in the epidemiology of schistosomiasis mansoni in an endemic area in Brazil. Bulletin of the World Health Organization. 1987;65(1):57.

45. Lima e Costa MFF, Rocha RS, Leite MLC, Carneiro RG, Colley D, Gazzinelli G, et al. A multivariate analysis of socio-demographic factors, water contact patterns and Schistosoma mansoni infection in an endemic area in Brazil. Revista do Instituto de Medicina Tropical de São Paulo. 1991;33:58-63.

46. Costa MFL, Rocha RS, Magalhães MHdA, Katz N. A hierarchical model for analysis of socio-economic variables and water contact patterns associated with the hepatosplenic form of schistosomiasis. Cadernos de Saúde Pública. 1994;10:S241-S53.

47. Enk MJ, Lima AC, Barros Hda S, Massara CL, Coelho PM, Schall VT. Factors related to transmission of and infection with Schistosoma mansoni in a village in the South-eastern Region of Brazil. Mem Inst Oswaldo Cruz. 2010;105(4):570-7.

48. Erismann S, Diagbouga S, Odermatt P, Knoblauch AM, Gerold J, Shrestha A, et al. Prevalence of intestinal parasitic infections and associated risk factors among schoolchildren in the Plateau Central and Centre-Ouest regions of Burkina Faso. Parasites & Vectors [Electronic Resource]. 2016;9(1):554.

49. Exum NG, Kibira SPS, Ssenyonga R, Nobili J, Shannon AK, Ssempebwa JC, et al. The prevalence of schistosomiasis in Uganda: A nationally representative population estimate to inform control programs and water and sanitation interventions. PLoS Negl Trop Dis. 2019;13(8):e0007617.

50. Farooq M, Nielsen J, Samaan S, Mallah M, Allam A. The epidemiology of Schistosoma haematobium and S. mansoni infections in the Egypt-49 project area: 2. Prevalence of bilharziasis in relation to personal attributes and habits. Bulletin of the World Health Organization. 1966;35(3):293.

51. Fentahun A, Hailu T, Alemu G. Prevalence of Intestinal Parasites and Schistosoma mansoni and Associated Factors among Fishermen at Lake Tana, Northwest Ethiopia. Biomed Res Int. 2021;2021:4534689.

52. Fentie T, Erqou S, Gedefaw M, Desta A. Epidemiology of human fascioliasis and intestinal parasitosis among schoolchildren in Lake Tana Basin, northwest Ethiopia. Trans R Soc Trop Med Hyg. 2013;107(8):480-6.

53. Fetene Y, Hailu T, Yimer M, Alemu M. Determinants of Helminthic Infections and Anemia among Schoolchildren in Bahir Dar Zuria District, Northwest Ethiopia. Journal of Parasitology Research. 2021;2021.

54. Firmo JO, COSTA MFLE, Guerra HL, Rocha RS. Urban schistosomiasis: morbidity, sociodemographic characteristics and water contact patterns predictive of infection. International journal of epidemiology. 1996;25(6):1292-300.

55. Fürst T, Ouattara M, Silué KD, N’Goran DN, Adiossan LG, Bogoch II, et al. Scope and limits of an anamnestic questionnaire in a control-induced low-endemicity helminthiasis setting in South-Central Côte d’Ivoire. PLoS One. 2013;8(6):e64380.

56. Ghazy RM, Ellakany WI, Badr MM, Taktak NEM, Elhadad H, Abdo SM, et al. Determinants of Schistosoma mansoni transmission in hotspots at the late stage of elimination in Egypt. Infect. 2022;11(1):102.

57. Gichuki PM, Kepha S, Mulewa D, Masaku J, Kwoba C, Mbugua G, et al. Association between Schistosoma mansoni infection and access to improved water and sanitation facilities in Mwea, Kirinyaga County, Kenya. BMC Infect Dis. 2019;19(1):503.

58. Gomes DS, Leal de Oliveira B, Silva Coelho PR, Mendonca Severino ADJ, Moreira Teodoro de Oliveira N, Thiengo SC, et al. Mapping Risk Factors and Spatial Clusters of Schistosomiasis Mansoni in Rural Communities from Brazil: Three Cross-Sectional Studies between 2014-2022. Ssrn. 2025;31.

59. Guimarães MD, Barros HLd, Katz N. A clinical epidemiologic study in a schistosomiasis mansoni endemic area (Tuparecê, Minas Gerais). Revista do Instituto de Medicina Tropical de São Paulo. 1985;27:123-31.

60. Guimarães MDC, Costa MFFdL, Lima LBd, Moreira MA. Clinical-epidemiological study of schistosomiasis mansoni in school children of Ilha, Arcos County, Minas Gerais, Brazil, 1983. Revista de Saúde Pública. 1985;19:8-17.

61. Guo XY, He N, Jiang QW. [Risk factors associated with reinfection of Schistosoma japonicum]. Zhonghua Liu Xing Bing Xue Za Zhi. 2004;25(8):691-4.

62. Hailegebriel T, Nibret E, Munshea A, Ameha Z. Prevalence, intensity and associated risk factors of Schistosoma mansoni infections among schoolchildren around Lake Tana, northwestern Ethiopia. PLoS Negl Trop Dis. 2021;15(10):e0009861.

63. Hailu T, Mulu W, Abera B. Effects of water source, sanitation and hygiene on the prevalence of schistosoma mansoni among school age children in Jawe District, Northwest Ethiopia. Iranian Journal of Parasitology. 2020;15(1):124-9.

64. Hailu T, Alemu M, Abera B, Mulu W, Yizengaw E, Genanew A, et al. Multivariate analysis of factors associated with Schistosoma mansoni and hookworm infection among primary school children in rural Bahir Dar, Northwest Ethiopia. Trop Dis Travel Med Vaccines. 2018;4(1).

65. Hilali AH, Madsen H, Daffalla AA, Wassila M, Christensen NO. Infection and transmission pattern of Schistosoma mansoni in the Managil irrigation scheme, Sudan. Ann Trop Med Parasitol. 1995;89(3):279-86.

66. Houmsou R, Kela SL, Suleiman MM, J.A O. Perceptions and assessment of risk factors in schistosoma haematobium infection in Buruku and Katsina-Ala Local Government Areas of Benue State-Nigeria. Internet Journal of Infectious Diseases. 2010;8.

67. Ibrahim T, Zemene E, Asres Y, Seyoum D, Tiruneh A, Gedefaw L, et al. Epidemiology of soil-transmitted helminths and Schistosoma mansoni: a base-line survey among school children, Ejaji, Ethiopia. J Infect Dev Ctries. 2018;12(12):1134-41.

68. Ito EE, Eze CN, Nduka FO, Balogun BJ, Babalola AS, Owhe-Ureghe UB. Multivariate approach to risk factors profiling of urinary schistosomiasis in Ase-Niger River catchment, Southern Nigeria. Sci African. 2025;27.

69. Jeza VT, Mutuku F, Kaduka L, Mwandawiro C, Masaku J, Okoyo C, et al. Schistosomiasis, soil transmitted helminthiasis, and malaria co-infections among women of reproductive age in rural communities of Kwale County, coastal Kenya. BMC Public Health. 2022;22(1):136.

70. Joof E, Sanyang AM, Camara Y, Sey AP, Baldeh I, Jah SL, et al. Prevalence and risk factors of schistosomiasis among primary school children in four selected regions of The Gambia. PLoS Negl Trop Dis. 2021;15(5):e0009380.

71. Kabatereine NB, Standley CJ, Sousa-Figueiredo JC, Fleming FM, Stothard JR, Talisuna A, et al. Integrated prevalence mapping of schistosomiasis, soil-transmitted helminthiasis and malaria in lakeside and island communities in Lake Victoria, Uganda. Parasites & vectors. 2011;4:1-14.

72. Kabuyaya M, Chimbari MJ, Manyangadze T, Mukaratirwa S. Schistosomiasis risk factors based on the infection status among school-going children in the Ndumo area, uMkhanyakude district, South Africa. Southern African Journal of Infectious Diseases. 2017;32:67-72.

73. Kajembe VR, Gasarasi DB, Tarimo DS, Lushina M, Sylvester B. Prevalence and factors associated with persistent transmission of Schistosoma haematobium among primary school children after five rounds of mass drug administration using praziquantel: A cross sectional study in Mkuranga district, Tanzania. Tropical Doctor. 2022;52(4):526-31.

74. Kim ES, Adriko M, Oseku KC, Lokure D, Webb EL, Sabapathy K. Factors associated with hookworm and Schistosoma mansoni infections among school-aged children in Mayuge district, Uganda. BMC Public Health. 2024;24(1):1620.

75. Knopp S, Stothard JR, Rollinson D, Mohammed KA, Khamis IS, Marti H, et al. From morbidity control to transmission control: time to change tactics against helminths on Unguja Island, Zanzibar. (Special Issue: CONTRAST alliance: optimized surveillance and sustainable control of schistosomiasis.). Acta Tropica. 2013;128(2):412-22.

76. Kulinkina AV, Kosinski KC, Adjei MN, Osabutey D, Gyamfi BO, Biritwum NK, et al. Contextualizing Schistosoma haematobium transmission in Ghana: Assessment of diagnostic techniques and individual and community water-related risk factors. Acta Tropica. 2019;194:195-203.

77. Kumagai T, Matsumoto-Takahashi ELA, Ishikawa H, Keomalaphet S, Khattignavong P, Soundala P, et al. Detection of Schistosoma mekongi DNA in Human Stool and Intermediate Host Snail Neotricula aperta via Loop-Mediated Isothermal Amplification Assay in Lao PDR. Pathogens. 2022;11(12):24.

78. Lakew A, Kibru G, Biruksew A. Prevalence of intestinal parasites among street beggars in Jimma town, Southwest Ethiopia. Asian Pacific Journal of Tropical Disease. 2015;5:S85-S8.

79. M'Bra RK, Kone B, Yapi YG, Silue KD, Sy I, Vienneau D, et al. Risk factors for schistosomiasis in an urban area in northern Cote d'Ivoire. Infectious Diseases of Poverty. 2018;7(1):47.

80. Mahmud MA, Spigt M, Mulugeta Bezabih A, Lopez Pavon I, Dinant G-J, Blanco Velasco R. Risk factors for intestinal parasitosis, anaemia, and malnutrition among school children in Ethiopia. Pathog Glob Health. 2013;107(2):58-65.

81. Malibiche D, Mushi V, Justine NC, Silvestri V, Mhamilawa LE, Tarimo D. Prevalence and factors associated with ongoing transmission of Schistosoma haematobium after 12 rounds of Praziquantel Mass Drug Administration among school age children in Southern Tanzania. Parasite Epidemiol Control. 2023;23:e00323.

82. Marçal Júnior O, Hotta LK, Patucci RMdJ, Glasser CM, Dias LCdS. Schistosomiasis mansoni in an area of low transmission: II. Risk factors for infection. Revista do Instituto de Medicina Tropical de São Paulo. 1993;35:331-5.

83. Masaku J, Njomo DW, Njoka A, Okoyo C, Mutungi FM, Njenga SM. Soil-transmitted helminths and schistosomiasis among pre-school age children in a rural setting of Busia County, Western Kenya: a cross-sectional study of prevalence, and associated exposures. BMC Public Health. 2020;20(1):356.

84. Maseke LS, Mushi V, Tarimo D, Kwesigabo G, Mazigo H. Adolescents and young adults excluded from preventive chemotherapy for schistosomiasis control in Northern Tanzania: are they at risk and reservoirs of infection? Prevalence and determinants of transmission in Northern Tanzania. IJID Regions. 2022;4:111-9.

85. Massara CL, Peixoto SV, Barros Hda S, Enk MJ, Carvalho Odos S, Schall V. Factors associated with schistosomiasis mansoni in a population from the municipality of Jaboticatubas, State of Minas Gerais, Brazil. Mem Inst Oswaldo Cruz. 2004;99(5 Suppl 1):127-34.

86. Mathewos B, Alemu A, Woldeyohannes D, Alemu A, Addis Z, Tiruneh M, et al. Current status of soil transmitted helminths and Schistosoma mansoni infection among children in two primary schools in North Gondar, Northwest Ethiopia: a cross sectional study. BMC Research Notes. 2014;7:88.

87. Matthys B, Tschannen AB, Tian‐Bi NT, Comoé H, Diabaté S, Traoré M, et al. Risk factors for Schistosoma mansoni and hookworm in urban farming communities in western Cote d'Ivoire. Tropical Medicine & International Health. 2007;12(6):709-23.

88. Mohammed T, Hu W, Aemero M, Gebrehiwot Y, Erko B. Current Status of Urinary Schistosomiasis Among Communities in Kurmuk District, Western Ethiopia: Prevalence and Intensity of Infection. Environ Health Insights. 2023;17:11786302231172323.

89. Mota E, Sleigh AC. Water-contact patterns and Schistosoma mansoni infection in a rural community in northeast Brazil. Rev Inst Med Trop Sao Paulo. 1987;29(1):1-8.

90. Moza PG, Pieri OS, Barbosa CS, Rey L. [Sociodemographic and behavioral factors related to schistosomiasis in a rural village of the sugar cane belt in Pernambuco State, Brazil]. Cad Saude Publica. 1998;14(1):107-15.

91. Munisi DZ, Buza J, Mpolya EA, Kinung'hi SM. Intestinal Schistosomiasis among Primary Schoolchildren in Two On-Shore Communities in Rorya District, Northwestern Tanzania: Prevalence, Intensity of Infection and Associated Risk Factors. Journal of Parasitology Research. 2016;2016.

92. Murenjekwa W, Makasi R, Ntozini R, Chasekwa B, Mutasa K, Moulton LH, et al. Determinants of Urogenital Schistosomiasis Among Pregnant Women and its Association With Pregnancy Outcomes, Neonatal Deaths, and Child Growth. J Infect Dis. 2021;223(8):1433-44.

93. Musuva RM, Odiere MR, Mwinzi PNM, Omondi IO, Rawago FO, Matendechero SH, et al. Unprotected water sources and low latrine coverage are contributing factors to persistent hotspots for schistosomiasis in western Kenya. PLoS ONE. 2021;16(9):e0253115.

94. Mutsaka-Makuvaza MJ, Matsena-Zingoni Z, Katsidzira A, Tshuma C, Chin'ombe N, Zhou XN, et al. Urogenital schistosomiasis and risk factors of infection in mothers and preschool children in an endemic district in Zimbabwe. Parasit Vectors. 2019;12(1):427.

95. N'Zi CK, Ouattara M, Assaré RK, Bassa FK, Diakité NR, N'Goran EK. Risk Factors and Spatial Distribution of Schistosoma mansoni Infection among Preschool-Aged Children in Blapleu, Biankouma District, Western Côte d'Ivoire. Journal of Tropical Medicine. 2021;2021.

96. Ndassa A, Mimpfoundi R, Gake B, Paul Martin MV, Poste B. Risk factors for human schistosomiasis in the Upper Benue valley, in northern Cameroon. Ann Trop Med Parasitol. 2007;101(6):469-77.

97. Ndokeji S, Mazigo HD, Temu M, Kishamawe C, Malenganisho W, Todd J, et al. Prevalence and intensity of schistosoma mansoni and hookworm infections among pre-school and school-aged children in Ilemela district, north-western Tanzania. Tanzania Journal of Health Research. 2016;18(2).

98. Ndyomugyenyi R, Minjas JN. Urinary schistosomiasis in schoolchildren in Dar-es-Salaam, Tanzania, and the factors influencing its transmission. Ann Trop Med Parasitol. 2001;95(7):697-706.

99. Ngui SM, Mwangangi JM, Richter J, Ngunjiri JW. Prevalence and intensity of urinary schistosomiasis and soil-transmitted helminths among women of reproductive age in Mwaluphamba, Kwale. Asian Pacific Journal of Tropical Medicine. 2024;17(2):71-83.

100. Nigo MM, Odermatt P, Salieb-Beugelaar GB, Morozov O, Battegay M, Hunziker PR. Epidemiology of Schistosoma mansoni infection in Ituri Province, north-eastern Democratic Republic of the Congo. PLoS Negl Trop Dis. 2021;15(12):e0009486.

101. Njambi E, Magu D, Masaku J, Okoyo C, Njenga SM. Prevalence of Intestinal Parasitic Infections and Associated Water, Sanitation, and Hygiene Risk Factors among School Children in Mwea Irrigation Scheme, Kirinyaga County, Kenya. Journal of Tropical Medicine. 2020;2020.

102. Noman MA, Alshargby SA, Kadi HO, Mansoor T, Rahman RA, Abudo GS, et al. Spread of internal parasites among the student of Al-Shaheed Al-Noman school, Widi Jadeed villages, Taiz province-Republic of Yemen. CJ BioMed. 2012;6:18-24.

103. Nworie O, Nya O, Anyim C, Okoli C, Okonkwo E. Prevalence of urinary schistosomiasis among primary school children in Afikpo North Local government area of Ebonyi State. 2012.

104. Nyirenda SS, Mooya LE, Mwansa JCL. Prevalence and risk factors associated with the transmission of urinary schistosomiasis among the school-going children in Kafue district, Zambia. Journal of Zoonotic Diseases. 2022;6(3):122-8.

105. Obadiah HI, Idu ME, Omudu EA, Shenge MF, Ameh MO, Mwakyoga A. Studies on schistosoma haematobium infection in school-aged children in some parts of Benue state, Nigeria. Niger J Parasitol. 2018;39(1):48-52.

106. Ojeleye FS, Bello ZU, Orakpoghenor O. Urinary schistosomiasis in pupils of Almajiri schools in Zangon-Shanu, Zaria, Kaduna State, Nigeria. Scientific Reports in Life Sciences. 2024;5(3):48-56.

107. Okoyo C, Campbell SJ, Williams K, Simiyu E, Owaga C, Mwandawiro C. Prevalence, intensity and associated risk factors of soil-transmitted helminth and schistosome infections in Kenya: Impact assessment after five rounds of mass drug administration in Kenya. PLoS Negl Trop Dis. 2020;14(10):e0008604.

108. Okoyo C, Campbell SJ, Owaga C, Onyango N, Medley G, Mwandawiro C. Statistical Regression Model of Water, Sanitation, and Hygiene; Treatment Coverage; and Environmental Influences on School-Level Soil-Transmitted Helminths and Schistosome Prevalence in Kenya: Secondary Analysis of the National Deworming Program Data. Am J Trop Med Hyg. 2021;104(6):2251-63.

109. Omondi I, Odiere MR, Rawago F, Mwinzi PN, Campbell C, Musuva R. Socioeconomic determinants of Schistosoma mansoni infection using multiple correspondence analysis among rural western Kenyan communities: Evidence from a household-based study. PLoS ONE. 2021;16(6):e0253041.

110. Onyekwere AM, Rey O, Nwanchor MC, Alo M, Angora EK, Allienne JF, et al. Prevalence and risk factors associated with urogenital schistosomiasis among primary school pupils in Nigeria. Parasite Epidemiology and Control. 2022;18.

111. Opara KN, Wilson EU, Yaro CA, Alkazmi L, Udoidung NI, Chikezie FM, et al. Prevalence, Risk Factors, and Coinfection of Urogenital Schistosomiasis and Soil-Transmitted Helminthiasis among Primary School Children in Biase, Southern Nigeria. Journal of Parasitology Research. 2021;2021.

112. Opoku-Kwabi D, Sevor B, Sarpong EA, Sam PK, Frimpong AA, Marfo PA, et al. Prevalence of schistosomiasis among school children at Esuekyir community in the Central Region of Ghana. BMC Infect Dis. 2024;24(1):1004.

113. Paller VGV, Belizario VY, Jr., Ancog RC, Alonte AJI, Jimenez JRD, Corales CG, et al. Socio-economic risk factors for intestinal helminthiases in selected endemic communities in Mindanao, the Philippines: a cross-sectional study. BMC Infect Dis. 2024;24(1):1012.

114. Palmeira DCC, de Carvalho AG, Rodrigues K, Couto JLA. Prevalence of Schistosoma mansoni infection in two municipalities of the State of Alagoas, Brazil. Revista da Sociedade Brasileira de Medicina Tropical. 2010;43(3).

115. Phillips AE, Mekete K, Firdawek E, Ower A, Maddren R, Anderson R, et al. Association between Water, Sanitation, and Hygiene Access and the Prevalence of Soil-Transmitted Helminth and Schistosome Infections in Wolayita Zone, Ethiopia. American Journal of Tropical Medicine and Hygiene. 2023;108(4 Supplement):366-7.

116. Phiri BBW, Ngwira B, Kazembe LN. Analysing risk factors of co-occurrence of schistosomiasis haematobium and hookworm using bivariate regression models: Case study of Chikwawa, Malawi. Parasite Epidemiology and Control. 2016;1(2):149-58.

117. Reitzug F, Kabatereine NB, Byaruhanga AM, Besigye F, Nabatte B, Chami GF. Current Schistosoma mansoni exposure and infection have distinct determinants: a data-driven population-based study in rural Uganda. medRxiv. 2024;24.

118. Reuben RC, Tanimu H, Musa J. Epidemiology of urinary schistosomiasis among secondary school students in Lafia, Nasarawa State, Nigeria. Epidemiology. 2013;3(2).

119. Risikat SA, Ayoade AA, editors. CORRELATION ANALYSIS BETWEEN THE PREVALENCE OF SCHISTOSOMA HAEMATOBUIM AND WATER CONDITIONS: A Case Study among the School Pupils in Southwestern Nigeria2012.

120. Rodrigues RN, Murta C, Júnior MACT, Cury GC, da Costa Rocha MO. Estudo clínico epidemiológico da esquistossomose mansoni no povoado de Ponte do Pasmado, município de Itinga, Minas Gerais (Brasil), 1992. Revista do Instituto de Medicina Tropical de São Paulo. 1995;37(1):81-5.

121. Rogers K, Ivan K, Eria T, Angella T, Ruth N, Immaculate N, et al. Human intestinal schistosomiasis and associated factors among children aged 5-15 years in Buyende district, eastern Uganda. Journal of Protozoology Research. 2024;34(1/2):14-24.

122. Rollemberg CV, Silva MM, Rollemberg KC, Amorim FR, Lessa NM, Santos MD, et al. Predicting frequency distribution and influence of sociodemographic and behavioral risk factors of Schistosoma mansoni infection and analysis of co-infection with intestinal parasites. Geospat Health. 2015;10(1):303.

123. Ross AG, Olveda RM, McManus DP, Harn DA, Chy D, Li Y, et al. Risk factors for human helminthiases in rural Philippines. International Journal of Infectious Diseases. 2017;54:150-5.

124. Ruganuza DM, Mazigo HD, Waihenya R, Morona D, Mkoji GM. Schistosoma mansoni among pre-school children in Musozi village, Ukerewe Island, North-Western-Tanzania: prevalence and associated risk factors. Parasit Vectors. 2015;8:377.

125. Rujeni N, Bayingana JB, Nyandwi E, Ntakarutimana A, Kagabo J, Rutayisire R, et al. Prevalence Mapping of Schistosoma mansoni Among Pre-school Age Children in Rwanda. Frontiers in Pediatrics. 2022;10.

126. Sady H, Al-Mekhlafi HM, Mahdy MA, Lim YA, Mahmud R, Surin J. Prevalence and associated factors of Schistosomiasis among children in Yemen: implications for an effective control programme. PLoS Negl Trop Dis. 2013;7(8):e2377.

127. Salawu OT, Odaibo AB. Schistosomiasis transmission; socio-demographic, knowledge and practices as transmission risk factors in pregnant women. Journal of Parasitic Diseases. 2016;40(1):93-9.

128. Samweli LG, Sesera AJ, Mushi V, Silvestri V, Palilo H, John W, et al. Intestinal schistosomiasis among secondary school students in Northern Tanzania: prevalence, infection intensity and associated risk factors. IJID Reg. 2023;6:125-34.

129. Schmidlin T, Hurlimann E, Silue KD, Yapi RB, Houngbedji C, Kouadio BA, et al. Effects of hygiene and defecation behavior on helminths and intestinal protozoa infections in Taabo, Cote d'Ivoire. PLoS ONE. 2013;8(6):e65722.

130. Sekre JK, Diakite NR, Assare RK, Kouadio JN, Coulibaly G, Konan CK, et al. Potential associations between Schistosoma mansoni infection and physico-chemical characteristics and water-related human activities in Cote d'Ivoire: a cross-sectional study. Parasit Vectors. 2024;17(1):422.

131. Shabani M, Zacharia A, Mushi V, Joseph M, Kinabo C, Makene T. Prevalence and predictors of intestinal schistosomiasis among the adult population, and water and sanitation conditions-A community-based cross-section study at Muleba District, Tanzania. Rwanda Med J. 2022;79(1):36-43.

132. Silva LK, Barbosa LM, Kovach JD, Dos Santos Teixeira R, Soares ES, Cardoso CW, et al. The changing profile of schistosomiasis in a changing urban landscape. International Journal for Parasitology. 2020;50(1):27-34.

133. Sitotaw B, Shiferaw W. Prevalence of Intestinal Parasitic Infections and Associated Risk Factors among the First-Cycle Primary Schoolchildren in Sasiga District, Southwest Ethiopia. Journal of Parasitology Research. 2020;2020.

134. Soares MS, Barreto MG, Silva CL, PA Cd, Pereira JB, Moza PG, et al. Schistosomiasis in a low prevalence area: incomplete urbanization increasing risk of infection in Paracambi, RJ, Brazil. Memórias do Instituto Oswaldo Cruz. 1995;90:451-8.

135. Sousa-Figueiredo JC, Stanton MC, Katokele S, Arinaitwe M, Adriko M, Balfour L, et al. Mapping of Schistosomiasis and Soil-Transmitted Helminths in Namibia: The First Large-Scale Protocol to Formally Include Rapid Diagnostic Tests. PLoS Negl Trop Dis. 2015;9(7):e0003831.

136. Stephenson LS, Elliot TC, Kinoti SN. Water, Sanitation and Knowledge about Urinary Schistosomiasis in a Kenyan Coastal Community: A study combining ethnographic and survey techniques. In: Stephenson LS, editor. Schistosomiasis and Malnutrition. Ithaca: Cornell University, Division of Nutritional Sciences; 1986. p. 69-192.

137. Stothard JR, Mgeni AF, Khamis S, Seto E, Ramsan M, Rollinson D. Urinary schistosomiasis in schoolchildren on Zanzibar Island (Unguja), Tanzania: a parasitological survey supplemented with questionnaires. Trans R Soc Trop Med Hyg. 2002;96(5):507-14.

138. Sudat SE, Carlton EJ, Seto EY, Spear RC, Hubbard AE. Using variable importance measures from causal inference to rank risk factors of schistosomiasis infection in a rural setting in China. Epidemiol Perspect Innov. 2010;7:3.

139. Surakat O, Alabi O, Ogundana T, Rufai A, Adeleke M, Sam-Wobo S, et al. Evaluation of Water Sanitation and Hygiene (WASH) Facilities and Its Association with Urinary Schistosomiasis in Selected Settlements of Osun State, Nigeria. Pan African Journal of Life Sciences. 2020;4:51-8.

140. Tadege B, Shimelis T. Infections with Schistosoma mansoni and geohelminths among school children dwelling along the shore of the Lake Hawassa, southern Ethiopia. PLoS ONE. 2017;12(7):e0181547.

141. Tadesse D, Kabeta L, Zewdneh T, Solomon K. Index of potential contamination for intestinal schistosomiasis among school children of Raya Alamata District, Northern Ethiopia. Momona Ethiopian Journal of Science. 2013;5(2):32-48.

142. Tanser F, Azongo DK, Vandormael A, Barnighausen T, Appleton C. Impact of the scale-up of piped water on urogenital schistosomiasis infection in rural South Africa. eLife. 2018;7(2):20.

143. Tazebew B, Temesgen D, Alehegn M, Salew D, Tarekegn M. Prevalence of S. mansoni Infection and Associated Risk Factors among School Children in Guangua District, Northwest Ethiopia. Journal of Parasitology Research. 2022;2022.

144. Tiruneh A, Kahase D, Zemene E, Tekalign E, Solomon A, Mekonnen Z. Identification of transmission foci of Schistosoma mansoni: narrowing the intervention target from district to transmission focus in Ethiopia. BMC Public Health. 2020;20(1):769.

145. Tolera B, Mohammed J, Umer U, Abamecha J, Tebeje F, Sime A, et al. Prevalence, intensity, and associated factors of intestinal schistosomiasis among primary school children in Nono District, Southwest Ethiopia. Front Trop Dis. 2024;5.

146. Tupps C, Kargbo-Labour I, Paye J, Dhakal S, Hodges MH, Jones AH, et al. Community-wide prevalence and intensity of soil-transmitted helminthiasis and Schistosoma mansoni in two districts of Sierra Leone. PLoS Negl Trop Dis. 2022;16(5):e0010410.

147. Umar A. The Prevalence and Intensity of Urinary Schistoso-miasis Among School Children Living along the. The Nigerian postgraduate medical journal. 2005;12(3):169.

148. Usman AM, Babeker EA. A study on the aspects of epidemiology of urinary and intestinal schistosomiasis in Bauchi State, Nigeria. Science World Journal. 2017;12(4):88-93.

149. Usman AM. Epidemiological studies of schistomiasis in bauchi central senatorial zone, Nigeria. Curr Trends Biotechnol Pharm. 2020;14(5):226-32.

150. Vonghachack Y, Odermatt P, Taisayyavong K, Phounsavath S, Akkhavong K, Sayasone S. Transmission of Opisthorchis viverrini, Schistosoma mekongi and soil-transmitted helminthes on the Mekong Islands, Southern Lao PDR. Infectious Diseases of Poverty. 2017;6(1):131.

151. Wanjala PM, Khaemba BM, Luoba AI. Prevalence and intensity of infection of intestinal schistosomiasis and reinfection after intervention in Budalangi endemic focus of Western Kenya. International Journal of Tropical Medicine. 2013;8:71-80.

152. Watts S, Katsha SE. Changing environmental conditions in the Nile delta: health and policy implications with special reference to schistosomiasis. International Journal of Environmental Health Research. 1995;5(3):197-212.

153. Wepnje GB, Anchang-Kimbi JK, Ndassi VD, Lehman LG, Kimbi HK. Schistosoma haematobium infection status and its associated risk factors among pregnant women in Munyenge, South West Region, Cameroon following scale-up of communal piped water sources from 2014 to 2017: a cross-sectional study. BMC Public Health. 2019;19(1):392.

154. Wubet K, Damtie D. Prevalence of Schistosoma mansoni Infection and Associated Risk Factors among School Children in Jiga Town, Northwest-Ethiopia: A Cross-Sectional Study. Journal of Parasitology Research. 2020;2020.

155. Ximenes R, Southgate B, Smith PG, Guimarães Neto L. Socioeconomic determinants of schistosomiasis in an urban area in the Northeast of Brazil. Revista Panamericana de Salud Publica. 2003;14(6):409-21.

156. Yahaya S, Auta T, Atalabi TE, Ahmed NY, Hassan J, Joshua EJ. Risk factors associated with urinary schistosomiasis and its spatial distribution among male children in selected communities in Shinkafi, Zamfara State, Nigeria. Dutse Journal of Pure and Applied Sciences. 2024;10(3b):29-37.

157. Yang J, Zhao Z, Li Y, Krewski D, Wen SW. A multi-level analysis of risk factors for Schistosoma japonicum infection in China. International Journal of Infectious Diseases. 2009;13(6):e407-e12.

158. Yangaza Y, Mushi V, Zacharia A. Prevalence of urogenital schistosomiasis and risk factors for transmission among primary school children in an endemic urban area of Kinondoni municipality in Dar es Salaam, Tanzania. Microbes and Infectious Diseases. 2022;3(1):230-40.

159. Yusuf AS, Moi IM, Hassan MA, Abubakar BM. Prevalence, associated risk factors and molecular identification of urinary schistosomiasis among primary school pupils in Jama'are Local Government Area, Bauchi State, Nigeria. Journal of Parasitic Diseases. 2025;(no pagination).

160. Zeleke AJ, Addisu A, Tegegne Y. Prevalence, Intensity, and Associated Factors of Schistosoma mansoni among School Children in Northwest Ethiopia. Journal of Parasitology Research. 2020;2020.
